# Autophagy gene haploinsufficiency drives chromosome instability, increases migration, and promotes early ovarian tumors

**DOI:** 10.1371/journal.pgen.1008558

**Published:** 2020-01-10

**Authors:** Joe R. Delaney, Chandni B. Patel, Jaidev Bapat, Christian M. Jones, Maria Ramos-Zapatero, Katherine K. Ortell, Ralph Tanios, Mina Haghighiabyaneh, Joshua Axelrod, John W. DeStefano, Isabelle Tancioni, David D. Schlaepfer, Olivier Harismendy, Albert R. La Spada, Dwayne G. Stupack

**Affiliations:** 1 UC San Diego Moores Cancer Center, La Jolla, California, United States of America; 2 Department of Obstetrics, Gynecology, and Reproductive Sciences, UC San Diego School of Medicine, La Jolla, California, United States of America; 3 Departments of Neurology, Neurobiology, and Cell Biology, and the Duke Center for Neurodegeneration & Neurotherapeutics, Duke University School of Medicine, Durham, North Carolina, United States of America; 4 Department of Biochemistry and Molecular Biology, Medical University of South Carolina, Charleston, South Carolina, United States of America; 5 Department of Pediatrics and Division of Biological Sciences, UC San Diego School of Medicine, La Jolla, California, United States of America; 6 Division of Biomedical Informatics, Department of Medicine, UC San Diego School of Medicine, La Jolla, California, United States of America; Brigham and Women's Hospital, UNITED STATES

## Abstract

Autophagy, particularly with *BECN1*, has paradoxically been highlighted as tumor promoting in Ras-driven cancers, but potentially tumor suppressing in breast and ovarian cancers. However, studying the specific role of *BECN1* at the genetic level is complicated due to its genomic proximity to *BRCA1* on both human (chromosome 17) and murine (chromosome 11) genomes. In human breast and ovarian cancers, the monoallelic deletion of these genes is often co-occurring. To investigate the potential tumor suppressor roles of two of the most commonly deleted autophagy genes in ovarian cancer, *BECN1* and *MAP1LC3B* were knocked-down in atypical (*BECN1*+/+ and *MAP1LC3B*+/+) ovarian cancer cells. Ultra-performance liquid chromatography mass-spectrometry metabolomics revealed reduced levels of acetyl-CoA which corresponded with elevated levels of glycerophospholipids and sphingolipids. Migration rates of ovarian cancer cells were increased upon autophagy gene knockdown. Genomic instability was increased, resulting in copy-number alteration patterns which mimicked high grade serous ovarian cancer. We further investigated the causal role of *Becn1* haploinsufficiency for oncogenesis in a MISIIR SV40 large T antigen driven spontaneous ovarian cancer mouse model. Tumors were evident earlier among the *Becn1*+/- mice, and this correlated with an increase in copy-number alterations per chromosome in the *Becn1*+/- tumors. The results support monoallelic loss of *BECN1* as permissive for tumor initiation and potentiating for genomic instability in ovarian cancer.

## Author summary

Life requires an excellent trash and recycling system. One system mammalian cells rely upon is called autophagy. Autophagy clears cells of larger forms of debris and is critical for normal cell function. We previously found that ovarian cancer cells are unable to execute normal cellular recycling during periods of stress, due to losses in core autophagy genes. Since autophagy has previously been implicated as a cancer cell survival factor, we directly investigated the impact of autophagy gene loss in ovarian cancer models. We discovered a reduction in autophagy proteins disabled normal genome quality control during cell division, leading to genomic instability. We document here for the first time whether reducing an autophagy gene in mice influences ovarian tumor formation. We found earlier ovarian tumors in autophagy deficient mice compared with control mice. We conclude that serous ovarian carcinomas, which rank among the most genetically altered cancers, gain their hallmark genomic instability in part by losing autophagy genes.

## Introduction

The role of autophagy in cancer remains enigmatic. The canonical cellular task of autophagy is to recycle damaged proteins, organelles, and fatty acids [1]. For protein quality control, autophagy is partially redundant and complementary to other cellular homeostasis pathways, such as the endoplasmic reticulum stress response and the ubiquitin-proteasome system. Due to its central role in recycling metabolites, especially in hypoxic tumor environments, autophagy has been demonstrated to aid in tumor progression [2]. The role of autophagy as a tumor promoting cellular pathway is particularly evident in Ras- or Raf-driven tumors. Complete deletion of *Atg7*, an essential autophagy gene, dramatically slows tumor growth in these mouse models [3, 4]. This is in part due to depleted pools of glutamine and glutamate preventing nucleotide synthesis [5]. However, autophagy has also been described as a tumor suppressor pathway. Its role in protein quality control affects multiple homeostatic mechanisms, many of which interact with DNA repair pathways [6–8]. BECN1, also known as beclin 1 [9], has been highlighted for its tumor suppressor roles ever since two independent labs generated heterozygous whole-body knockout mouse models; each study observed earlier cancer formation in *Becn1*+/- mice compared to *Becn1*+/+ mice [10, 11].

Tumor suppressor gene deletions require additional modulators to form cancer. In human breast cancer and ovarian cancer *BECN1* is almost always co-deleted with *BRCA1*. This led to the assumption that *BECN1* loss is a passenger event and is only deleted due to its proximity to *BRCA1* [12]. Recent experiments and genomic analyses have suggested that moderate tumor suppressors are actually co-deleted with adjacent or distant tumor suppressors during cancer evolution, such as those near *TP53* on chromosome 17 [13]. For these *TP53*-co-deletions, some, such as *EIF5A* and *ALOX15B*, act in an independent fashion to suppress tumor formation. *BRCA1* deletions similarly involve *BECN1*. In human breast cancers, reduced expression of *BECN1* at the RNA level indicated a poor prognosis [14]. Re-expression of *BECN1* in a heterozygous deleted *BECN1* human breast cancer cell line MCF7 reduced clonogenicity in soft agar [15]. Aside from *BECN1*, few haploinsufficient models of autophagy have been studied in the context of cancer.

Our prior bioinformatic studies suggest that monoallelic deletions in autophagy genes are unusually pervasive in high-grade serous ovarian cancer (SOC) (98% of tumors have multiple heterozygous deletions), although these heterozygous deletions are present in many other cancer types. Homozygous deletions are exceedingly rare (<1% of genes in any tumor type) [16]. We previously found that monoallelic deletions in autophagy genes suppressed the ability of ovarian cancer cells to overcome proteotoxic stress. These deletions similarly limited autophagy induction. Upon autophagic stress, ovarian cancer cells selectively perished without the need for programmed cell death via apoptosis or necroptosis, resulting in a low-toxicity treatment [17]. Considering that completely compromising autophagic flux via homozygous deletion of autophagy genes can prevent tumor formation, it was unclear why tumors might arise while lacking so many autophagy alleles. One possibility is that dysregulation of this homeostatic pathway involved in DNA repair could lead to genomic instability. Heterogeneity has been linked to poor patient outcome [18]. Notably, SOC has among the greatest intra-tumor clonal heterogeneity in a pan-cancer analysis [19].

To investigate the effects of *BECN1* haploinsufficiency in ovarian cancer, we developed a pseudo-haploinsufficiency knockdown model using a human ovarian cancer cell line that is atypically autophagy-competent. *BECN1* or *MAP1LC3B* gene suppression resulted in increases in copy-number genomic instability. Extending these studies in a murine spontaneous ovarian cancer model, we found that tumors initiate earlier in *Becn1+/-* mice relative to *Becn1+/+* littermates. *Becn1+/-* murine tumors and autophagy suppressed human ovarian cancer cell lines displayed greater rates of chromosomal aberrations.

## Results

### Genetic context of *BECN1* deletions

**Fig 1:**
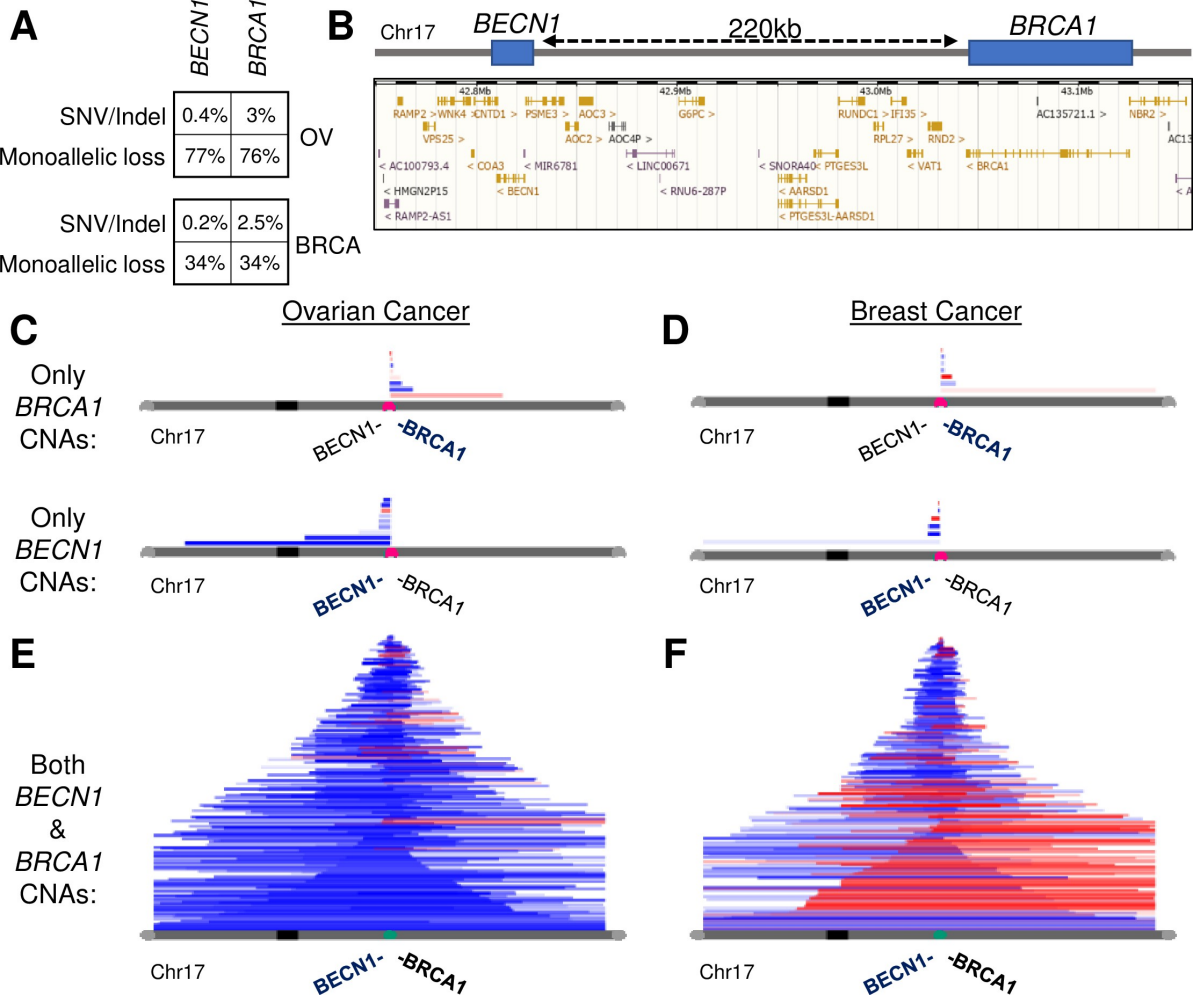
CAIRN analysis of co-alteration of *BECN1* and *BRCA1*. **A,** Single-nucleotide variants or insertion deletion rates for the adjacent genes *BECN1* and *BRCA1* are compared for the SOC (OV) and breast cancer (BRCA) datasets. Monoallelic loss rates are shown for comparison. **B,** Ensembl display of genomic region on Chr17 encompassing *BECN1* and *BRCA1*. **C-F**, A visualization tool CAIRN was developed to enable the oncology community to easily analyze and display copy-number alterations in human datasets (see Materials). Each horizontal segment is from an individual tumor and displays a continuous CNA of the chromosome. Blue segments represent copy-losses, red segments indicate copy-gains, and both are displayed in relation to the parent chromosome indicated in grey. Human TCGA tumors were tested for CNAs overlapping the genes *BECN1* and *BRCA1*. Ovarian tumors with exclusive *BRCA1* or *BECN1* deletions **(C)** are far rarer than tumors deleted in both genes (**E)** as shown by CAIRN. Breast tumors followed a similar pattern **(D,F)**. In both tumor types, *BECN1* deletions without accompanying *BRCA1* deletions were found.

Since the vast majority of genetic deletions in solid tumors are monoallelic, we queried The Cancer Genome Atlas (TCGA) data for mutations and heterozygous losses in *BECN1* and *BRCA1* using cBioPortal [20] and PanCancer Atlas data. In both SOC (OV) and breast cancer (BRCA), the frequency of monoallelic deletions of either *BECN1* or *BRCA1* was >10-fold that of the frequency of single-nucleotide variant or insertion-deletion mutations (Fig 1A). This is consistent with the hypothesis that monoallelic deletions predominantly act to reduce the expression levels of tumor suppressor genes [21, 22], rather than to achieve a loss-of-heterozygosity event complementing a point mutant. The concordance of *BRCA1* deletions and *BECN1* deletions between tumor types is expected from their close proximity on chromosome 17 (Fig 1B). The significance of exclusive deletions of only *BECN1* in human breast and ovarian cancer has been debated [12, 14]. However, to directly evaluate whether single copy-number alteration (CNA) events overlapped both genes, further bioinformatic analysis was undertaken.

### CAIRN: A tool to easily visualize and quantify copy-number alterations

The genetic lesions causing CNAs often overlap two or more tumor suppressor genes and oncogenes [21]. To readily visualize these changes, we created a bioinformatic tool to enable researchers without a bioinformatics background to readily test for gene co-deletion or co-amplification. The tool, “Copy Alterations Intuitive-Rendering Navigator” (CAIRN), permits stacked visualization of genetic gain or loss events not unlike the similarly-named stacked stone markers. CAIRN works with custom CNA datasets, queries of chromosome regions, and gene-centric queries. It can output segments which overlap the region(s) of interest or output segments which end at a region of interest (for example, telo-centric CNAs). The tool is available online at https://delaney.shinyapps.io/CAIRN/.

Data from 33 TCGA-PanCancer Atlas tumor types were uploaded into the public CAIRN database. We validated the program using the *PTK2* and *MYC* oncogenes, which are known to be co-amplified on Chromosome 8 [23]. Oncogenes on the same locus may co-promote tumor initiation and progression. Co-amplification CNAs of these oncogenes were prevalent in ovarian and breast cancer (S1 Fig). Quantitative analysis paired with the qualitative visualization of the CNAs by CAIRN will be useful for chromosome-localized gene pairs or gene sets.

It is believed that tumor deletion CNAs are selected to encompass multiple tumor suppressors (Fig 1D and 1E) [21]. A query for CNAs overlapping *BECN1* and/or *BRCA1* in ovarian cancer and breast cancer confirmed that *BECN1* is occasionally exclusively deleted (no co-deletion of *BRCA1*, 8 of 594 tumors for ovarian cancer and 4 of 1085 for breast cancer). *BRCA1* is occasionally exclusively deleted even less frequently (3 of 594 for ovarian cancer and 4 of 1085 for breast cancer) (Fig 1C and 1D). However, the vast majority of deletion segments are deleted for both (361 of 594 for ovarian cancer and 277 of 1085 for breast cancer), as might be expected from the hypothesis that tumor deletion CNAs are selected to encompass multiple tumor suppressors (Fig 1D and 1E) [21]. In summary, *BECN1* is found to be deleted independently of *BRCA1* in some ovarian and breast tumors, although the majority delete *BECN1* and *BRCA1* concomitantly.

### Clinical and genetic characteristics associated with autophagy gene loss

More than 95% of human ovarian cancers have deletions in 4 or more autophagy genes [16]. These include those encoding beclin 1 and the autophagosome biogenesis and cargo recruitment protein MAP1LC3B (simply, LC3B). LC3B is used as a reporter in the majority of cellular assays to measure quality and quantity of autophagy [9], and *LC3B* is heterozygous deleted in ~68% of SOC tumors and ~56% of breast tumors (S2 Fig). It is noteworthy that these occur as heterozygous deletions >99% of the time, not homozygous deletions, so a residual level of autophagy persists. To address the possibility that these deletions arise in specific patient populations, those patients with *BECN1* or *LC3B* tumor deletions were queried for clinical characteristics. Gene loss was not associated with race (S3A Fig), stage (S3B Fig), age (S3C Fig), nor somatic mutation burden (S3D Fig). However, the loss of either *BECN1*, *LC3B*, or both was associated with a higher percentage of CNA alterations within the tumor (S3E Fig). While individual gene loss did not confer significantly better or worse response to standard of care (S3F Fig), suppression of gene expression conferred slightly worse prognosis for *BECN1* (S2G Fig, *P <* 0.02) and a trend toward worse prognosis for *LC3B* (*P <* 0.067). Overall survival was also significantly worse in patients with a low HAPTRIG autophagy pathway score (S3H Fig, *P <* 0.043) [16]. The results suggest that the increased overall autophagy gene loss in low scoring tumors, relative to higher scoring tumors, impacts patient outcomes.

### Autophagy in ovarian cancer cells knocked-down in *BECN1* and *LC3B*

**Fig 2:**
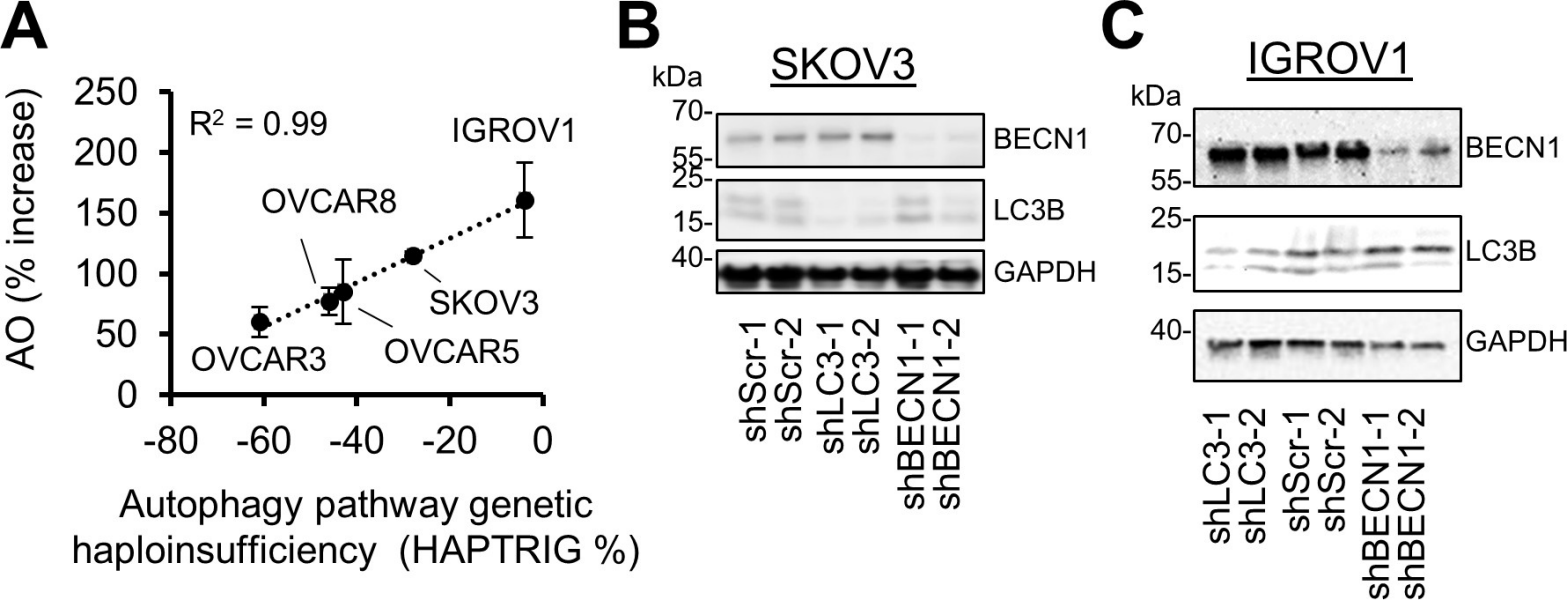
Titrated knockdown of *BECN1* or *LC3B* reduces autophagosome accumulation markers. **A,** Commonly used ovarian cancer cell lines were tested for acidic vacuole accumulation following chloroquine treatment (10μM 24h) by flow cytometric measurement of acridine orange (AO). Shown are the averages of 4 flow cytometry assays. The x-axis depicts a pathway network calculation from HAPTRIG defining the levels of autophagy pathway haploinsufficiency within each cell line due to monoallelic losses of KEGG autophagy pathway genes. **B,** SKOV3 cells, which do not have losses in *BECN1* nor *LC3B*, were stably knocked-down by shRNA lentivirus. A western blot was performed for the targets LC3B and BECN1, along with GAPDH loading control. **C,** IGROV1 cells were similarly assayed.

Among the ovarian cancer cell models available for study, no high-grade serous ovarian cancer cell lines have a perfectly diploid level of all autophagy genes. This complicates the selection of cell lines to study autophagy, as it is technically infeasible to revert to diploid each one of 4–12 lost autophagy alleles in high-grade serous ovarian cancer cell lines. To define appropriate models to study suppression of *BECN1* or *LC3B*, a flow cytometric analysis of acridine orange accumulation in widely used cell lines was performed. Acridine orange becomes protonated and unable to pass through lipid membranes when it enters an acidic compartment such as lysosomes, autophagic vacuoles, and amphisomes [9]. In parallel, the level of autophagy genetic suppression in each cell line was predicted by HAPTRIG [16], which uses cell line gene deletion information, protein-protein interaction data, and haploinsufficiency phenotypes. A strong correlation (R^2^ = 0.99) between acidic compartment accumulation and HAPTRIG-predicted suppression of autophagy genes was found (Fig 2A), suggesting monoallelic changes predictably contribute to the disruption of organelles essential for autophagy.

The cell lines best able to accumulate acidic vacuoles were IGROV1 and SKOV3 cells. IGROV1 is a hyper-mutated mixed lineage cell line with a Y126C p53 mutation. SKOV3 is a histologically serous cell line which does not express p53 [24]. Neither cell line contains deletions in *BECN1* nor *LC3B* (S4 Fig), permitting their use in controlled autophagy gene knockdown studies. To phenocopy haploinsufficiency, we selected shRNAs targeting *BECN1* and *LC3B* whose expression resulted in intermediate reduction of protein expression. We focused on two shRNAs whose impact was neither complete nor minimal, but as close to a 50% target as possible within the suite of shRNAs we tested [16]. These shRNAs were confirmed to moderately knock down their target gene in SKOV3 (Fig 2B) and IGROV1 (Fig 2C) cells.

The direct manipulation of *LC3B* complicates the study of autophagic flux. Commonly used flux assays involve observing the conversion of LC3-I to LC3-II or introducing a tagged (RFP-)GFP-LC3B. The latter method confounds the interpretation of effects since the tagged-LC3B would be predicted to rescue shRNA knockdown. The former is complicated since autophagic measurements compare LC3B to a control protein. To study acidic recycling compartments independent of individual autophagy or lysosomal proteins, an acridine orange staining assay was performed. This does not measure autophagic flux specifically [9], but rather turnover of all acidic organelles within the cell. SKOV3 cells were blocked from autolysosome resolution using chloroquine, induced for autophagy using the mTORC1 inhibitor rapamycin, or both (S5A Fig). As expected, SKOV3 cells knocked-down in *BECN1* or *LC3B* both exhibited significantly reduced acidic punctae staining relative to scrambled control shRNA in all conditions (S5B Fig). Similar results were found using IGROV1 cells (S5C and S5D Fig). This result contrasts with previous findings in which knockdown of *BECN1* does not necessarily reduce LC3 lipidation [25]. In summary, autophagy markers and acidic organelle recycling were decreased in SKOV3 and IGROV1 cells in both the shBECN1 and shLC3B conditions.

### Oncogenic phenotypes from autophagy gene knockdown

**Fig 3:**
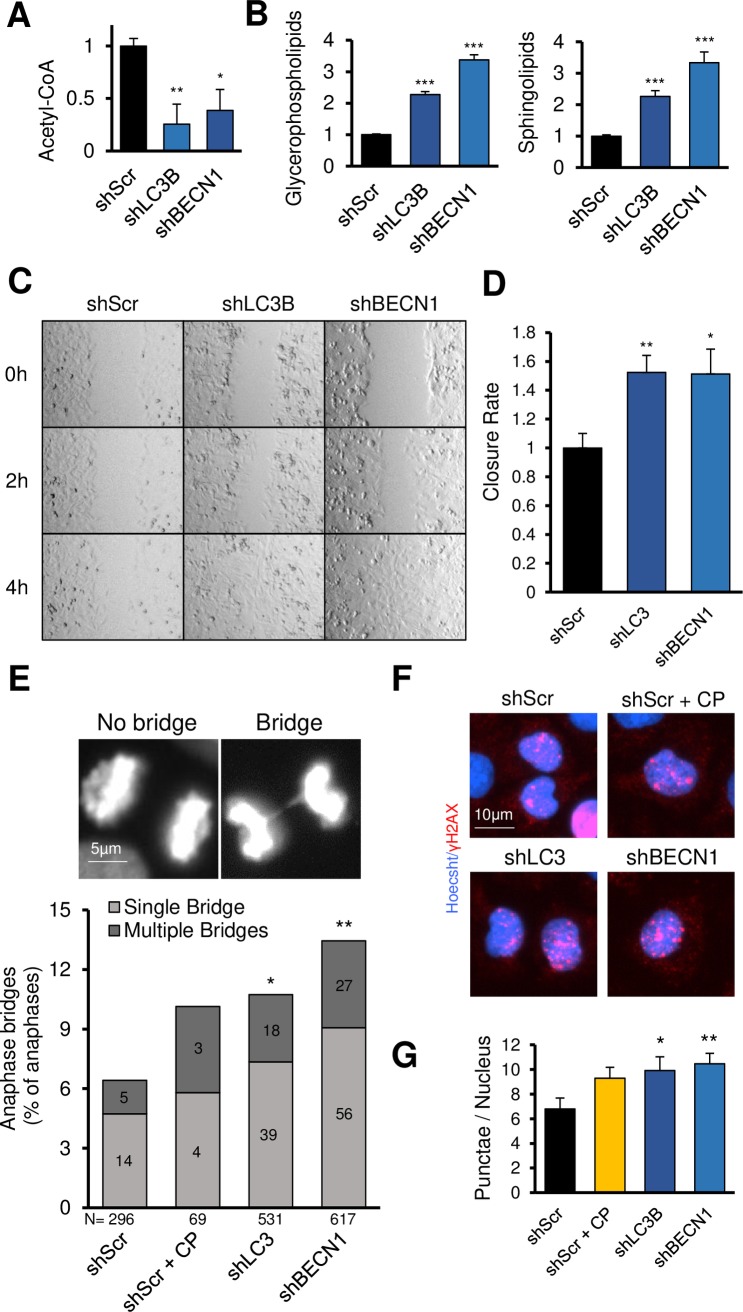
Knockdown of *LC3B* or *BECN1* increases oncogenic phenotypes. **A-B,** SKOV3 cells transduced by the indicated lentivirus were assayed by metabolomics. Quantitation of the indicated metabolites is shown. **C,** Confluent SKOV3 cells were tested for migration rates using a scratch-wound assay. **D,** Quantitation of three independent wound closure experiments. **E,** Dividing SKOV3 cells were imaged for DNA content, blinded, and scored for anaphase bridge formation. CP denotes cisplatin use. N is the number of anaphases analyzed from three independent assays. **F,** SKOV3 cells were imaged for γH2AX puncta. N > 900 cells per condition from two independent assays. **G,** Quantitation of (F). * indicates *P ≤* 0.05, **P ≤ 0.01, ***P ≤ 0.001.

We next evaluated potential roles for *BECN1* and *LC3B* in suppressing known oncogenic phenotypes. Since autophagy is a catabolic pathway, metabolomics were performed on knockdown cells to determine alterations in metabolism by ultra-performance liquid chromatography mass-spectrometry (UPLC-MS/MS) (S6A and S6B Fig). The most significant changes were found in lipid metabolism. Acetyl-CoA levels were reduced to 26% or 39% of control in shLC3B and shBECN1 cells, respectively (Fig 3A). This may result from an increase in anabolic lipid metabolism, as a concomitant increase of 2.2–3.7-fold was observed for glycerophospholipids and sphingolipids (Fig 3B). These lipids are required for new membrane formation during cellular proliferation, and glycerophospholipid synthesis is proposed as a drug target for cancer [26]. Canonical energy signaling molecules ATP, ADP, AMP, and cAMP were largely unchanged (S6C Fig), with the exception of a 13% increase of ADP in shLC3B cells. A reduction in free amino acids may be predicted from a reduction of autophagy, yet this was not observed in SKOV3 cells (S6D Fig). Putricine levels were 2.7 times shScr levels in shLC3B cells, while downstream metabolites spermidine and spermine levels were elevated but not significantly increased (S6E Fig). CoA (S6F Fig), NAD+, and both oxidized and reduced forms of glutathione were unchanged. (S6G Fig). Thus, autophagy gene suppression altered lipid metabolism but not canonical energy metabolites.

In parallel, we tested a CRISPR-Cas9 total knockout of *LC3B* (S6H Fig). Notably, the total disruption of this autophagy gene yielded a metabolism distinct from the impaired but functioning system in the shLC3B and shBECN1 cells (S6I and S6J Fig). This finding is consistent with the notion that dose-dependent decreases in autophagy yields a markedly different phenotype than a total knockout, which provides one explanation as to why homozygous loss rarely occurs in tumors.

Autophagy is implicated in focal adhesion turnover and cell migration via recruitment of paxillin to LC3 or by recruitment of the selective autophagy cargo receptor NBR1 [27, 28]. Only marginal changes were observed in IGROV1 migration, which exhibits poor motility (S7A and S7B Fig), Notably, the migration-competent SKOV3 cells showed a marked acceleration of motility upon *LC3B* (52% faster) or *BECN1* knockdown (51% faster) (Fig 3C and 3D).

Genomic instability is a hallmark of cancer [29], with SOC specifically containing some of the highest rates of chromosome instability of any cancer. To determine if genomic instability, and resultant heterogeneity, was altered among cells with deficient autophagy, SKOV3 cells were tested for an increase in stochastic DNA lesions, in particular ssDNA and dsDNA breaks, by alkaline comet assay [30]. However, no differences in tail moments were observed in autophagy knockdown SKOV3 cells (S7C Fig), suggesting a grossly similar incidence of lesion occurrence.

Defective cell division can lead to aneuploidy. We reasoned that since *BECN1* or other autophagy gene loss may promote centrosome instability [31], our shBECN1 and shLC3B cells may contain an unusual complement of centrosomes. Surprisingly, most cells had a single centrosome spot. No cell contained three distinct centrosomes (N > 100 cells per condition), nor were differences in centrosome size observed (S7D Fig). However, anaphase bridge formation occurred twice as frequently among autophagy deficient cells (Fig 3E). This was independent of BRCA1 level, since BRCA1 expression was constant among autophagy competent or deficient cells (S7E Fig). γH2AX foci were similarly increased in shLC3B and shBECN1 SKOV3 cells (Fig 3F and 3G), but not in IGROV1 cells (S7 Fig). Foci levels in shLC3B and shBECN1 cells were comparable to cisplatin-induced stress. Taken together, decreases in BECN1 or LC3B protein are sufficient to manifest genomic instability and increased migration, two hallmarks of aggressive tumors.

### Autophagy gene knockdown increases chromosome instability

**Fig 4:**
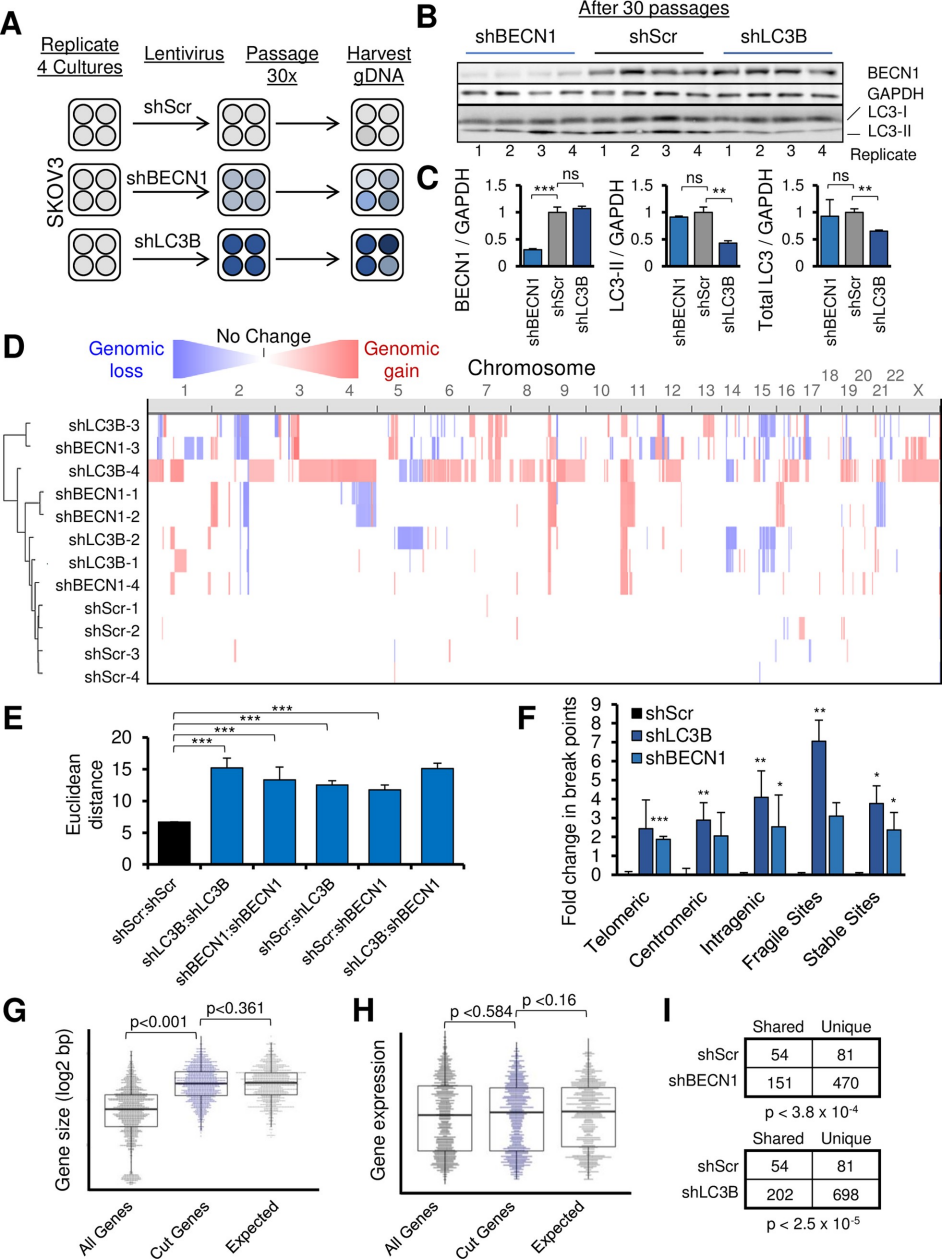
Spontaneous copy-number evolution in ovarian cancer cells. **A,** SKOV3 cells were stably knocked-down by shRNA lentivirus. Control, shBECN1, and shLC3B cultures were passaged 30 times (1:3 dilutions) with no selective pressure other than transgene selection. Genomic DNA was then harvested. **B,** SKOV3 remained knocked-down for target protein expression at the completion of the experiment. For early passage knockdown levels, please refer to Fig 2 and S6 Fig. **C,** Quantitation of western blot data. LC3-II is separately quantified from total LC3 to provide independent estimates of knockdown efficiency and autophagosomal LC3. **D,** Copy-number alteration profiles of SKOV3 transduced with shBECN1 or shLC3B, arranged by chromosome on the x-axis as indicated. Red coloration depicts copy-number gains while blue coloration depicts copy-number losses, relative to shScr controls. Hierarchical clustering of copy-number data was performed, resulting in separation of autophagy-knockdown cells from controls. **E,** Euclidian distance metrics of copy-number alterations is compared between the genotypes. All autophagy knockdowns were more dissimilar from one another than the scrambled controls. **F,** Common break points normally found in tumor samples were compared to break points of copy-number changes in SKOV3 cells. **G-H,** CNA edges of SKOV3 cells suppressed in autophagy (shBECN1 and shLC3B combined) were overlaid with genes and analyzed for **(G)** gene size and **(H)** expression level. While expression level did not predict gene breakage, larger genes were more often disrupted by CNA break points. However, this would be expected by chance, as shown by 1,000 randomizations of the observed CNAs (“Expected” plot). **I,** Fisher’s exact test tables comparing CNA segments that are shared between knockdown cells and scrambled control cells. * indicates *P ≤* 0.05, **P ≤ 0.01, ***P ≤ 0.001, ns *P >* 0.05.

To quantify whether DNA-bridging and γH2AX foci contributed to genomic instability in SKOV3 cells, we transduced and passaged four populations of SKOV3 cells for 30 passages in a genetic drift assay (Fig 4A). After 30 passages, to our surprise, there was no increased expression of LC3B or beclin 1 in our cells. Thus, under standard tissue culture conditions, there is no overt selection pressure to restore autophagy despite documented metabolomic changes (Fig 4B and 4C). High resolution Oncoscan arrays (>220,000 genomic targets) were used to assess de novo CNAs [32]. For statistical purposes of comparing experimental to control samples, the reduced segment method was used [33]. Unbiased hierarchical clustering of these CNAs separated shBECN1 and shLC3B from shScr controls (Fig 4D). The Euclidean distance of these CNAs, which is a measure of genetic drift, was higher between all autophagy-suppressed pairs than variation amongst the controls (Fig 4E). The greater genetic drift between autophagy-suppressed samples was evident independent of normalization to the control. Since both focal and megabase scale changes were observed, we tested whether the edges of these CNAs originated from known unstable DNA regions in cancer cells (see Methods for references). Interestingly, *BECN1* knockdown cells were enriched for telomeric and intragenic breaks (Fig 4F), while *LC3B* knockdown cells were enriched in centromeric, intragenic, and fragile site breaks. However, it is noteworthy that “stable” chromatin also had elevated rates of CNA formation in both autophagy gene knockdown groups, suggesting breaks were not explained only due to chromosomal fragility.

*BECN1* knockout can result in gross aneuploidy in multiple model systems [34, 35]. To evaluate more subtle copy-number changes, we annotated the CNAs by chromosome location and base-pair length according to genotype. Subtle CNA variation was observed for shScr control SKOV3 cells across the genome for focal (<100kb) CNAs, but was rare for large (>1Mb) CNAs. There was a significant increase in both focal and large CNAs in shBECN1 cultures (S8A Fig). However, some chromosomes were more unstable than others (S8B Fig). *LC3B* knockdown exhibited similar disruption of both focal and large CNAs. Shared alterations included telomeric 9p (amplified in 7 of 8 shBECN1 and shLC3B cultures), chromosome arm 11p (amplified in 7 of 8 shBECN1 and shLC3B cultures), and centromeric chromosome 2 (deletions in 8 of 8 shBECN1 and shLC3B cultures). Deletions in chromosome arm 14p were shared across shLC3B cultures but not shBECN1 cultures, as were 15q deletions. In 3 of 4 shBECN1 cultures but only 1 of 4 shLC3B cultures, deletions within 21q were observed (summarized in S1 Table).

Common break sites lay within exceptionally long genes [36]. The patterns of CNA breaks and regions were checked for repetitive patterns and random effects. Since we identified an increase in intragenic dsDNA breaks in our SKOV3 autophagy-compromised cells, we wondered if the same pattern was observed. While very few previously identified genes were disrupted by CNA formation in our evolved SKOV3 cells (S1 Table), we did observe a similar bias toward increased length, but not expression, in genes harboring genomic breaks (Fig 4G and 4H). To investigate whether this was significant, we performed a permutation analysis to determine if simply by having completely random CNAs of the same size as we observed in our samples, similar results would be found. In fact, randomized CNAs of equal length to the observed CNAs were observed more frequently on large genes relative to the actual distribution of gene sizes in the human genome (Fig 4G and 4H). Therefore, our observations are not explained by genomic breaks which occur during the transcriptional regulation of long or active genes. To further evaluate the level of randomness, reduced segment CNAs were compared between shScr cells and autophagy gene knockdown cells. The ratio of single-sample unique CNAs to shared (potentially non-random, found in at least two samples) CNAs was found to be 1.5 in shScr isolates (Fig 4I), while in shBECN1 this ratio was 3.1 (Fisher’s exact *P <* 3.8 x 10^−4^) and 3.5 in shLC3B (*P <* 2.5 x 10^−5^). Randomness was not different between shBECN1 and shLC3B cultures (*P* < 0.42). Thus, while there are more overall shared segments within autophagy gene knockdown cells, there is an even greater increase in randomly changed CNAs.

### Pathway analysis of CNAs

To determine patterns that were consistently altered in the shBECN1 and shLC3B cells compared to controls, we used two pathway analysis tools: HAPTRIG [16] and GSEA [37]. GSEA is a commonly used tool to measure curated sets of genes which are altered at statistically improbable rates, whereas HAPTRIG further incorporates interaction and haploinsufficiency data to generate pathway network scores, outperforming GSEA at identification of tumor suppressors and oncogenes using cancer CNA data [16]. Here, KEGG pathway sets were used, since KEGG is one of the few pathway sets containing “autophagy” as a discrete pathway. We have reasoned that if the suppression of autophagy confers an adaptive selection event, individual gene deletion may not be as important as the ultimate functional impact on the autophagy pathway. Indeed, among patients the genes deleted within the autophagy pathway vary widely from tumor to tumor.

**Fig 5:**
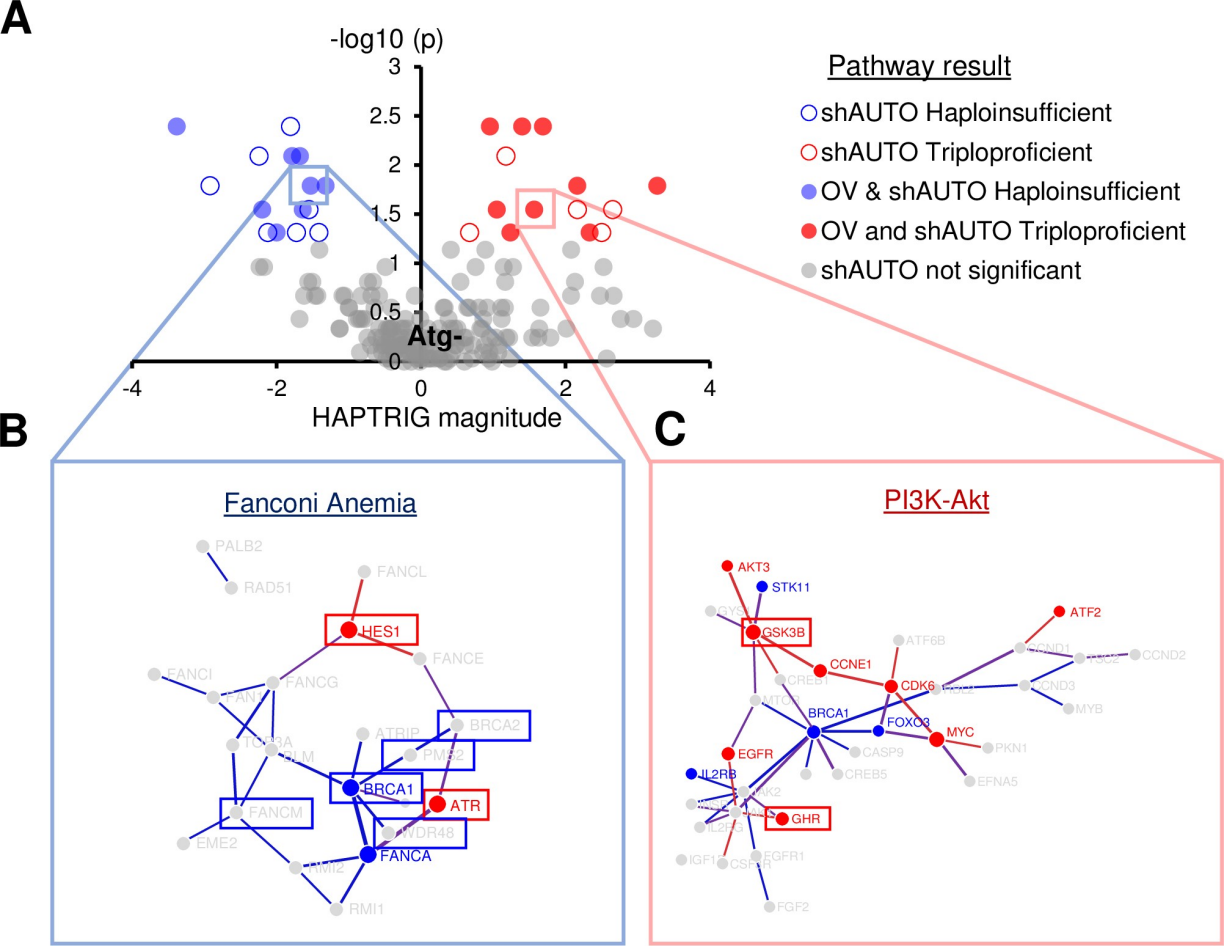
CNA patterns affect SOC pathways. **A,** HAPTRIG pathway analysis of SKOV3 copy-number alterations. Nominally significant (*P <* 0.05) pathways enriched for suppression or elevation of CNAs in SKOV3 autophagy suppressed cells (shBECN1 and shLC3B combined, “shAUTO”) by the gene-set analysis tool HAPTRIG are displayed as red (elevated) or blue (suppressed). Open colored circles indicate pathways only found in SKOV3 cells, and closed colored circles indicate pathways also found to be similarly dysregulated in TCGA studied serous ovarian cancer primary tumors (OV). **B-C,** Select altered pathways from TCGA serous ovarian cancer data are highlighted and networks were graphed by HAPTRIG. Scores were tabulated and those gene nodes with scores one z-score away from the median node score are displayed with blue if suppressed and in red if elevated. Edges indicate physical interactions as found in BioGrid. Boxes were drawn around genes if HAPTRIG found this node to be one of the top five influential genes (suppression and elevation were separately tested) within the selected pathway for both the SKOV3 experiment and TCGA ovarian cancers.

Similar to how tumor data is analyzed, results from shBECN1 and shLC3B knockdown cultures were pooled as a cohort (“shAUTO”) and compared to shScr control cultures. HAPTRIG analysis identified 29 differentially altered pathways in shBECN1/shLC3B cultures relative to shScr, at a significance of *P* ≤ 0.05 as appropriate to the small sample size. Notably, many of the differentially altered pathways in SKOV3 shAUTO cells were altered concordant with that observed human ovarian tumors studied by the TCGA (filled circles in Fig 5A). Fanconi-anemia, RNA degradation, regulators of NOTCH signaling, and TNF signaling were all allelically suppressed (Fig 5A; S1 Table). Differentially altered pathways that were copy-number upregulated included the glycerolipid metabolism pathway, which was previously found as one of the most upregulated pathways in SOC [16]. Endocytosis, PI3K-Akt signaling, and central carbon metabolism in cancer were upregulated, further recapitulating pathways upregulated in human ovarian cancer. Complementary GSEA identified by *P* < 0.05 the copy-number upregulation of glycolysis, along with autophagy, toll-like receptor signaling, and cytosolic DNA sensing. HAPTRIG was used to tabulate which genes contribute the most to pathway alterations. Many of the same gene CNA hubs in human ovarian tumor networks are marked as hubs in the autophagy deficient cohort (shAUTO, Fig 5B). Taken together, these results indicate that moderate suppression of single autophagy genes can result in a strikingly altered genotype. Passaging the cells in culture without any phenotypic selection unexpectedly yielded pathway-level alterations that recapitulate those observed in human disease.

### Haploinsufficient *Becn1* ovarian cancer mouse model yields early tumors

While co-deletion of *BECN1* with the well-established tumor suppressor *BRCA1* occurs in the vast majority of *BRCA1* deletion CNAs (Fig 1E), *BECN1* has not been directly tested as a tumor suppressor in the context of ovarian cancer. Given the role of autophagy in suppressing chromosomal instability, it was not unreasonable that *BECN1* might act as a tumor suppressor. We investigated this using a spontaneous mouse model of ovarian cancer which recapitulates the inactivation of p53, a characteristic of the aggressive serous histotype [38–40]. The SV40 large T antigen driven by the MISIIR promoter inactivates p53 and other cell cycle regulators [41] in the ovarian and fallopian epithelium [42]. Mice expressing this transgene begin to exhibit ovarian tumors around 16–22 weeks of age.

**Fig 6:**
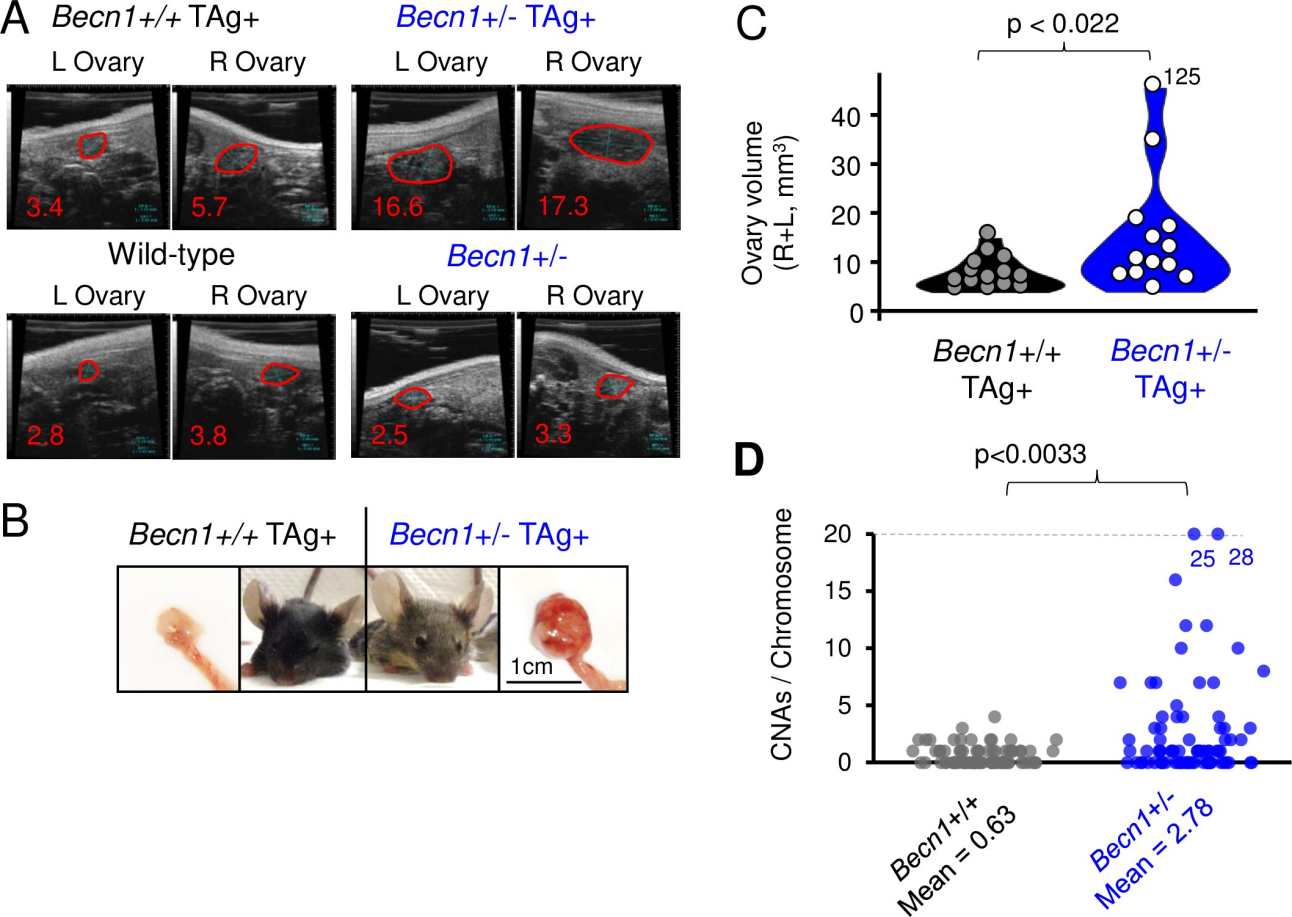
*Becn1*+/- mice present early ovarian tumors. **A,** Ultrasound images of tumor size at 12 weeks of age in the MISIIR Large-T-Antigen (TAg+) ovarian cancer mouse model. Example tumors for each genotype are shown for both left (L) and right (R) ovaries. Non-TAg+ controls are shown for comparison. **B,** Example 12-week tumors are shown and juxtaposed with an image of the mouse model, with characteristic large ears (TAg model) and brown fur coat (*Becn1*+/- model). **C,** Quantitation of all mice studied for ovarian size at 12 weeks of age. N = 13 for *Becn1*+/- TAg+, N = 14 for *Becn1*+/+ TAg+ mice. Wilcoxon rank-sum test was used to determine the *P-*value. **D,** Terminal ovarian tumors were isolated for genomic DNA and assayed by whole-genome sequencing. *Becn1*+/- tumors displayed higher rates of CNAs per chromosome. Two outlier chromosomes are labeled for CNA counts beyond the edge of the y-axis. N = 4 mice per group for whole-genome copy-number analysis; each data point represents one chromosome. Wilcoxon rank-sum test was used to determine the *P-*value.

We crossed *Becn1* heterozygous knockout females (*Becn1*+/-) with TAg males. The resulting litters are a mix of the expected four genotypes, and we compared female "BTAg" (*Becn1*+/- MISIIR SV40 Large T Antigen) mice to littermate "TAg" (*Becn1*+/+ MISIIR SV40 Large T Antigen) females. We performed ultrasounds on a cohort of mice for ovary size at 12 weeks: a time point in which TAg mice have not been observed to form tumors. Ultrasound size measurements indicated significant hypertrophy of ovaries in *Becn1*+/- TAg mice, but not in *Becn1*+/+ TAg controls (Fig 6A–6C). Early tumor initiation was confirmed by dissection and harvest of part of the cohort (Fig 6B), suggesting that impaired autophagy promotes or supports tumor initiation. Despite earlier tumors and a trend toward early morbidity, no significant difference in morbidity was observed between *Becn1*+/+ and *Becn1*+/- groups as the remaining mice were aged (S9 Fig), possibly as a result of insufficient cohort size. Large tumors, ascites, or ruptured ovaries were observed in 100% of the euthanized mice. Genomic instability (Fig 4) provided a possible mechanism for enhanced tumorigenesis in BTAg mice. To test if the tumors forming in BTAg mice had higher rates of chromosome instability, we dissected ovaries from mice requiring euthanasia from the morbidity study. We collected adjacent uterine tissue as a normal genomic control. Dissected samples were then processed to purify genomic DNA and whole-genome sequenced to low-pass coverage by NGS. Copy-number alterations present in each chromosome for each sample were then calculated and tabulated. No deletion of *Brca1* was observed. (S9 Fig, S3 Table). Overall rates of CNAs were low for these TAg murine tumors (median of 1 CNA per chromosome), despite histology that is otherwise similar to high grade serous ovarian cancer [42]. Nonetheless, BTAg mice had significantly higher CNAs per chromosome than control TAg mice (2.78 vs 0.68, *P <* 0.003 by Wilcoxon rank-sum test) (Fig 6D, S10 Fig). Overall, loss of one allele of *Becn1* enhanced tumor initiation and correlated with increased chromosomal alterations *in vivo*.

## Discussion

This study is the first to directly test the role of *Becn1* haploinsufficiency in an ovarian cancer oncogenesis model. Our controlled cell-culture models of suppressed autophagy in ovarian cancer demonstrated increases in cellular migration, DNA copy-number instability, and genomic evolution. Reduction of the dose of a single autophagy gene recapitulated differentially altered pathways which are common in serous ovarian cancer [43]. Monoallelic deletion of *Becn1* in an ovarian cancer model accelerated tumorigenesis in mice, while autophagy gene knockdown SKOV3 human ovarian cancer cells exhibited reduced autophagy gene expression, cellular migration rates increased, γH2AX signaling was elevated, and genomic stability compromised. Instability arose from breakage-fusion-bridge events but not increased DNA lesions nor centrosome abnormalities. Analysis of the evolved copy-number genotypes implicated an increase in the PI3K-Akt pathway and decreases in the Fanconi-anemia pathway, RNA degradation, Notch signaling, and TNF signaling, mimicking pathways impacted in human serous ovarian cancer.

### Autophagy acts as a tumor suppressor in ovarian cancer

Although loss of *BECN1* is purported to be solely a passenger mutation "piggybacking" on a *BRCA1* deletion during tumorigenesis [12], we directly observed formation of earlier ovarian tumors in *Becn1*+/- MISIIR-TAg+ mice compared to *Becn1*+/+ MISIIR-TAg+ mice. Context is important [44]. *BECN1* is co-deleted with *BRCA1* in over 95% of cases. *BRCA1* has been readily established as a tumor suppressor through epidemiological, mutational, and knockout mouse studies [45]. Previously reported findings suggesting that some cancers have only *BRCA1* deleted but not *BECN1* deleted, fail to hold true with increased growth and precision of the database of tumor CNAs. *BECN1* was found to be deleted alone without *BRCA1* deletion in both breast and ovarian human tumors (Fig 1). The catch-all statement that "*BECN1* … is not a tumor-suppressor gene" [12] is not supported by the current study.

### Physiological changes to cells upon autophagy gene suppression

Autophagy is a central metabolic homeostasis mechanism for cells. We tested through unbiased metabolomics of lipids and energy molecules for changes upon autophagy gene knockdown. While many aspects of metabolism remained largely unchanged, such as NAD+ and ATP, a severe (61–74%) depletion of acetyl-CoA was observed. This correlated with an increase in lipid content of the cells, which may aid in cell division or membrane mobility during migration. In addition to these metabolic changes, cellular migration was enhanced in SKOV3 cells reduced in either *BECN1* and *LC3B* levels. High grade serous ovarian cancer, which often originates within fallopian fimbriae, may require migration to the ovarian epithelium prior to formation of invasive carcinomas [46, 47]. A suppression of autophagy may facilitate such a migration.

Some phenotypic changes may be specific to the genetic or epigenetic environment surrounding autophagy gene knockdown. This is common in cancer studies evaluating differing tissue types [22]. Gain-of-function studies of mutant p53 suggest genomic instability to be dependent on the p53-interactome [48]. The observed γH2AX foci in SKOV3 cells but not IGROV1 cells may be due to the null-expression of p53 in SKOV3 cells, although further testing is required to evaluate this possibility. The large T-antigen expressed in the spontaneous mouse model sequesters p53 and is hypothesized to result in a null-p53 phenotype, among other tumor suppressors [41]. Phenotypes may be exacerbated by other mutations, such as the homozygous deletion of *CDKN2A* in SKOV3 cells, or the presence of an activating *PIK3CA* H1047R mutation. Interpretation of the current study may be limited in scope to epithelial ovarian tumors with a lack of p53 expression, such as the approximately one-third of serous ovarian cancers with nonsense or frameshift *TP53* mutations [49].

### The role of haploinsufficiency

Homozygous deletions are exceedingly rare in tumor cells (<1% of genes, compared to 25–80% of genes which are 1N or 3N in serous ovarian tumors). Serendipitously, *Becn1* has only been studied for haploinsufficient tumor suppressor roles *in vivo* due to the early embryonic lethality of homozygous deletion [11]. *Becn1* heterozygous deficient mice exhibit defects in autophagy, but no altered rates of apoptosis [10, 11], though *Becn1* haploinsufficiency compromises chromosomal stability [34]. A key difference between previous studies which showed tumor suppressor roles and those which showed oncogenic roles of autophagy is remarkably consistent: tumor suppressor roles are supported by evidence from heterozygous knockout or shRNA suppression models, whereas oncogenic roles are supported by homozygous knockout models. In the MMTV-*Wnt1* model, *Becn1*+/- mice progress much faster; median survival was 4 mo compared to *Becn1*+/+ mice at 7.2 mo [50]. Future studies which claim oncogenic roles of autophagy should include at least one model which does not require homozygous essential autophagy gene knockouts.

### Genomic instability following autophagy suppression

It is reasonable to conclude from our studies that normal dosage of *BECN1* and *LC3B* both enable genomic stability. Following an initial stimulus of *BECN1* or *LC3B* reduction. we detected widespread focal and large CNAs which were *BECN1*-specific as well as associated with *LC3B*. Human serous ovarian cancer CNAs partially derive from foldback-inversions: copy-number errors which occur following a breakage-fusion-bridge cycle [51]. The passaged SKOV3 autophagy gene knockdown cells indicated many telomeric break points (Fig 2E), consistent with foldback-inversions. Most genotoxic stresses induce autophagy through direct or indirect mechanisms. It is tempting to speculate that compromised autophagy (and mitophagy) simply results in an accumulation of defective mitochondria. The resultant released reactive oxygen species [52] would promote ssDNA and dsDNA breaks [53]. However, particularly in the case of *BECN1*, other possibilities exist. BECN1 is implicated in location specific roles modulating kinetochore and centromeric protein maintenance [35], which may explain why some autophagy markers do not change upon *BECN1* suppression [25], despite the observed functional phenotypes. Proteins which interact with BECN1 are enriched for chromosome organization and sister chromatid cohesion proteins (S2 Table).

It may be unexpected to find any phenotypes in cell line models containing shLC3B, since the LC3 family include three orthologs to *LC3B*: *LC3A*, *LC3B2*, and *LC3C*. The GABARAP, GABARAPL1, and GABARAPL2 proteins also contain amino acid sequence homology. Both LC3 family and GABARAP family proteins bind many of the same targets, including p62/SQSTM1 and NBR1 [54]. Hence, a modest reduction in just one of four LC3 orthologs may not be predicted to have any phenotype. However, there is evidence that LC3B punctae do not overlap LC3A punctae, whereas LC3A and LC3C punctae do overlap, suggesting LC3B may have unique and non-redundant functions in cancer cells [55]. While it is beyond the scope of the current study, evaluating the molecular and phenotypic differences between LC3 family members in the context of cancer is warranted. LC3 family interaction networks identified numerous unique targets which have been validated to have autophagy-independent roles [56]. Surprisingly, our data show that modest reduction of *LC3B* yields oncogenic phenotypes in ovarian cancer, similar to reduction of *BECN1*.

## Methods

### Ethics statement

All animal protocols were approved by the IACUC of University of California San Diego (S05356), of Duke University (A225-17-09), and of Medical University of South Carolina (00639).

### Cell culture, cell lines, and reagents

Established cell lines were purchased from the American Type Culture Collection (ATCC) and validated by short tandem repeat (STR) profiling (Promega). Routine microscopic morphology tests were performed prior to each experiment. Cells were verified to be mycoplasma negative by a PCR assay (Agilent Technologies (Stratagene), cat# 302008). All cells were grown in RPMI (Life Technologies) supplemented with 2% glucose, nonessential amino acids (Mediatech #45000–700), sodium pyruvate (Mediatech #45000–710), antibiotics (penicillin, streptomycin, and amphotericin, Mediatech #30-004-CI), and 10% FBS (Omega Scientific #FB-11). For all shRNA experiments, puromycin (VWR #IC1005521) was added to 1μg/ml in RPMI. Cells were cultured at 37°C with 5% CO_2_. *Antibodies*. LC3B (Novus Biologicals #NB100-2220), BECN1 (SantaCruz sc-11427), GAPDH (GeneTex #239), BRCA1 (ABclonal #A0212), and DyLight secondary (1:15,000 dilution) antibodies were used: 800nm for anti-rabbit (VWR #PI35571) and 680nm for anti-mouse (VWR # PI35518). Secondary HRP antibodies were anti-rabbit (Jackson ImmunoResearch #211-032-171), or anti-mouse (Jackson ImmunoResearch #115-035-003). spCas9 deletion of *MAP1LC3B* was performed using TTCAAGCAGCGCCGCACCTT sgRNA in a PX459 backbone. *Knockdown shRNAs*. Knockdowns for *MAP1LC3B* and *BECN1* were purchased from Thermo Fisher Scientific (#RHS4533-EG8678). Two shRNAs were always used to generate the presented figures: TRCN0000033550 (CCGACTTGTTCCTTACGGAAA) and TRCN0000033552 (CTCAAGTTCATGCTGACGAAT) for shBECN1, TRCN0000153286 (GAGTAGAAGATGTCCGACTTA) and TRCN0000155850 (CGCACCTTCGAACAAAGAGTA) for shLC3B.

### Mouse models

TgMISIIR‑TAg‑DR26 mice were a generous gift from the Connolly laboratory [42]. *Becn1*+/- mice originated from JAX laboratories, stock #018429, B6.129X1-Becn1^tm1Blev/J^, thanks to a deposition by Beth Levine. To generate *Becn1*+/- MISIIR TAg mice, female *Becn1*+/- dams were mated with MISIIR TAg sires. All experiments were performed with littermates and genotypes were confirmed by PCR. All animal protocols were approved by the IACUC of UCSD, and all rules and regulations were followed during experimentation on animals. The ultrasound experiment was powered to detect differences of 30% (http://homepage.divms.uiowa.edu/~rlenth/Power/). After genotyping, the mortality cohort of 13 TAg and 20 BTAg mice was predicted to have 78% power to detect 15% change in median survival with a 15% standard deviation. Ultrasound was performed in the UCSD Moores Cancer Center mouse imaging core, using a high resolution Vevo 2100 (FUJIFILM VisualSonics Inc). No blinding was performed, since mice were visually distinguishable by lighter fur coat color. All mice were female. Euthanasia criteria for the mortality experiments included loss of 20% of body weight, impairment of gait which prevented feeding or water consumption, or visible distension of abdomen indicating ascites.

### Western blotting

Cells were grown to 80% confluency on 10cm plates at 37°C. Media was aspirated, cells washed in PBS, and the cellular monolayer was immersed in iced RIPA buffer (supplemented with a protease inhibitor cocktail (Sigma-Aldrich), 2 mM sodium orthovanadate, and 50 mM NaF). Following 15 minutes at room temperature (to better extract LC3 [9]), lysates were collected using a cell lifter (Fisher Scientific). Lysates were spun at 10,000g for 10 minutes at 4°C and supernatant saved and quantified by BCA assay (Pierce #23235). 10–30μg protein was loaded per well of a 15% SDS-PAGE gel and transferred onto PVDF membrane. The membrane was blocked in 5% dry milk (Genesee Scientific, #20–241). Primary antibodies were used at 1:1000 dilution, and secondary HRP antibodies were used at 1:5,000 dilution or secondary fluorescent antibodies were used at 1:15,000. Fluorescent secondary antibodies were visualized using a LI-COR Odyssey scanner. Quantitation of band intensity was performed in ImageJ and all normalizations were to the loading control displayed in the corresponding figure.

### Flow cytometry

Acridine orange staining was performed identically as previously described [16]. HAPTRIG scores used for comparison were generated from CCLE copy-number alterations downloaded from the UCSC Xena Browser, as previously described using default HAPTRIG settings [16].

### Microarray analysis of copy-number alterations

Human SKOV3 cell lines were assayed for copy-number alterations using an Affymetrix OncoScan array. CEL files were processed by the Affymetrix chromosome analysis suite according to manufacturer’s instructions. Ploidy was verified and normalized in part by the known single allele of chromosome 7 and homozygous deletion of *CDKN2A*. Segmentation was performed from within the Affymetrix analysis suite. Since SKOV3 already contains CNAs and our experimental question was whether or not spontaneous CNAs formed in this model, we used the reduced segment method in CNTools (V1.32.0) [57] to create regions of differential copy-numbers. The mean segment amplitude of all four SKOV3 shScr replicates was used as the “normal” reference in the experiment, and all new CNAs were called if the magnitude was at least 0.2 units deviant from the normal.

### Sequencing analysis of murine copy-number alterations

Mice were euthanized according to the tumor burden requirements elucidated in the survival analysis section. Tumors were immediately resected, imaged, and snap frozen in liquid nitrogen. Tumors were stored in a -80°C freezer. For DNA extraction, tumors were individually thawed and processed by a Qiagen Blood and Tissue DNA kit, according to manufacturer’s instructions. Uterus tissue was removed and physically cleared of any tumor tissue and identically processed as normal adjacent tissue controls. DNA was then processed by the University of California San Diego Institute for Genomic Medicine sequencing core, according to normal Illumina protocols. DNA was sequenced for low-pass whole-genome coverage on an Illumina HiSeq4000 (~4Gb per sample) at the IGM Genomics Center, University of California, San Diego, La Jolla, CA. Paired-end reads were mapped to reference mm10 genome using Bowtie2. Mapped reads were input into HMMcopy (V1.18.0) [58], along with mappability and GC correction files as instructed by the HMMcopy package. Default settings were used to determine CNAs compared to normal tissue controls. Normal tissue was found to be essential in accurately determining segmented regions; without normal tissue controls many false positives are observed across all samples. A custom R script was written to produce the graphical outputs shown in S10 Fig.

### Quantitation of break site features

A custom R script was written to compare the distance of CNA edges observed in the SKOV3 cells to known features on the hg19 reference genome. These features include fragile and stable DNA sites from [59]. Regions within 2Mb of telomeres and centromeres as annotated by the UCSC Genome Browser were considered telomeric or centromeric breaks. Gene locations were annotated as from the first exon to then end of the last exon, including introns. Comparisons to randomized segments were performed by randomly moving equal sized segments of the observed segments on iso-chromosomal locations for 1,000 permutations and outputs included overlapping gene size or expression. Expression values were pulled from the CCLE dataset of the SKOV3 sample, downloaded from the UCSC Xena Browser.

### CAIRN database and accessibility

CAIRN was developed with the intent to distribute an easy-to-use tool for scientists with limited or no bioinformatic background to analyze copy-number variation around a locus of interest. It is available to use online for free at https://delaney.shinyapps.io/CAIRN/. For more complex or systematic use of the tool, code is available by download from https://github.com/jrdelaney/CAIRN. The Shiny R App is included within the zip package and installation and usage instructions are included within the zip file in pdf format. All included CNA data for default cancers online are from TCGA white-listed samples with segment information downloaded from the Genomic Data Commons: the PanCanAtlas summary (https://gdc.cancer.gov/about-data/publications/pancanatlas). Custom human data is enabled by uploading into the App or into the online version. Currently coordinates are set to hg38, but can be changed by altering files from the downloaded App.

### Clinical and genetic characteristics analysis

Tumor stage, racial group, age at diagnosis, and number of somatic mutations were taken directly from TCGA summary spreadsheets downloaded from cBioPortal [20]. Genome altered (%) was calculated from TCGA gene-level summary CNA data wherein only genes were considered, not intergenic regions or acrocentric chromosome arms (eg, 13p). Percent genome altered is thus based on the percent of genes with alterations, not the percent of the length of chromosomes altered. Survival plots were generated using KmPlot [60], since the number of samples exceeds TCGA data. For RNA expression cutoffs, the automatic cutoff selection criterion was used. For autophagy pathway-level Kaplan-meier analysis, HAPTRIG calculations of the lowest scored tertile was plotted against the highest scored tertile using survival and survminer R software packages.

### Fluorescence microscopy

For acridine orange staining, 10,000 cells were seeded in wells in a 96-well tissue-culture plate (VWR, #10062–900) in 100μL phenol-red free RPMI (Fisher Scientific, #11835030) media containing 4μg/mL puromycin. Media was not supplemented with non-essential amino acids nor sodium pyruvate. The day of imaging, 10μM chloroquine phosphate (VWR, #AAJ64459-14), 10nM rapamycin (LC Labs, R-5000), both, or control vehicle were added to the cells. Four hours later, acridine orange (Fisher Scientific, #AAL1315906) was added to 0.3μg/mL. Cells stained in the tissue culture incubator for 30 minutes prior to imaging. The plate was then imaged in the Texas Red channel in a BioTek LFX microscope using a 20X objective. For quantitation, ImageJ was used. The “Find maxima” function was used to identify acridine orange punctae, using individually selected cells. Two assays were performed and at least 100 cells from multiple fields quantified per condition.

For anaphase bridge counting, cells with stable integration of shScr, shBECN1, or shLC3B were seeded at 25,000 cells per well in a Nunc Lab-Tek II Chambered Coverglass, 8-well chamber slide (Thermo Scientific, #155409). For anaphase bridge assays, cells were grown for two days on the chamber slide, media was aspirated, 500μL of PBS slowly added and then aspirated, and 4% paraformaldehyde added for 15 minutes at room temperature. Cells were then washed once with PBS and stained for 30 minutes with Hoescht 33342 (1μg/mL in PBS). Cells were washed twice in PBS and mounted in 20μL VectaShield Antifade Mounting Medium (Vector Laboratories, #H-1000). Slides were imaged on a Nikon Eclipse 80i with a 40X objective or a BioTek LFX with a 20X objective and then pictures were blinded for anaphase bridge scoring analysis. Statistical comparisons were made using a Fisher’s Exact test.

For gamma-tubulin staining, cells were grown and processed similarly, but additionally permeabilized for 2 minutes with 0.1% Triton X-100 in PBS, blocked with 5% BSA / 5% goat serum for 30 minutes, stained with 488-conjugated γ-Tubulin (Fisher Scientific, # NB11090616X) for 1 hour RT and 16 hours 4°C, and washed 3x 30 minutes in PBS prior to mounting with VectaShield. Slides were imaged were imaged on a Nikon Eclipse 80i with a 40X objective.

For γH2AX staining, cells were grown to ~80% confluence. For cisplatin treated cells, cells were treated 48 h prior to fixation (13μM for SKOV3). Cells were washed with 50 μL PBS and then aspirated. Cells were fixed in 50μL of 4% PFA in PBS for 10 minutes and then 50 ul of 0.1% Triton X-100 in PBS for 2 minutes. The cells were washed again in 50 μL PBS. To block, 50μL of 5% bovine-serum-albumin (BSA, VWR #97061–416) and 5% goat-serum (GS, VWR #102643–594) in PBS was added to the cells and rocked for 45 minutes at room temperature. Supernatant was aspirated and 50μl of primary antibody solution (anti-H2AX phospho-Ser139 (Biolegend #613402) diluted 1:1000 with 5% BSA/GS in PBS) was added and rocked overnight at 4°C. The primary antibody solution was removed, and the cells were washed with 50μl PBS once followed by three additional 5-minute PBS washes. The cells were stained in 50μL of secondary antibody solution (Alexa Fluor 647 goat anti-mouse (Invitrogen #A21236) diluted 1:1000, Hoechst 33342 diluted 1:10,000, 5% BSA/GS in PBS), for 1.5 hours. The secondary antibody solution was removed, and cells were washed with one quick wash, three 5-minute washes, and one 30-minute PBS wash. Cells were imaged on a BioTek LFX microscope using DAPI and CY5 filters at 20x magnification, automatic focus, and equal exposure time. For analysis, FIJI was used [61]. Within each nucleus, the maxima function was used to identify punctae and the measure function analyzed each puncta’s intensity.

For cellular migration assays, shScr, shBECN1, shLC3B plasmids were transduced into SKOV3 and IGROV1 cells. Cells were selected with 2μg/ml puromycin. Cells were seeded into a 24-well tissue culture plate for IGROV1 (800k cells) and SKOV3 (600k cells) to reach confluence after 24-48h of growth. A P-10 pipette tip was used to create scratch wound. Images were captured every 2 hours for 24–48 hours on the BioTek LFX microscope and the area of the scratch wound was quantified using ImageJ. The percent of the wound remaining was calculated by dividing the area of the scratch at a given time point by the area at time 0. Subtracting the percent remaining from 100% yielded the percent closure at each time point. To obtain a relative rate, calculations divided by shScr rates.

### Comet assays

Cells were processed according to manufacturer’s protocol in the CometAssay Kit (Trevigen, #4250-050-K) using Alkaline preparation conditions. Staining was performed using SYBR Green 1X (from 10,000X stock, Thermo Fisher #S7563). Lysed nuclei comets were imaged on a Nikon Eclipse 80i at 40X and analyzed by OpenCOMET [62] for percentage of DNA in the tail moment.

### Metabolomics

All samples were grown to 80% confluency on a 10cm tissue culture dish. Cells were harvested by trypsinization and neutralized with iced RPMI complete media. Cells were washed twice in iced PBS and split into two tubes. Cell pellets were saved at -80°C until analysis. All sample sets had three independent cell growth experiments performed on different days.

For amino acid and lipid analysis, tubes containing ovarian cancer cell pellets were thawed at room temperature and then stored on ice during manipulation. For normalization, a duplicate pellet was analyzed by BCA assay for total content determination. 100 μL of 80/20 v/v MeOH/water was added to each sample tube. Samples were then probe sonicated 3 times at power level 3 for 5 seconds each burst, cooling on ice between bursts. Samples were then allowed to incubate for 10 minutes while on ice and then put in -80°C freezer until ready for analysis.

Samples were prepared using the AbsoluteIDQ p180 kit (Biocrates Innsbruck, Austria) in strict accordance with their detailed protocol. Samples were taken from the -80°C freezer and centrifuged at 4°C for 10 minutes at 15,000g. After the addition of 10 μL of the supplied internal standard solution to each well of the 96-well extraction plate, 15 μL of each ovarian study sample was added to the appropriate wells. The plate was then dried under a gentle stream of nitrogen for 10 minutes. An additional 15 μL of each study sample was added to the respective wells and plate was dried under nitrogen for an additional 20 minutes. The samples were derivatized with phenyl isothiocyanate then eluted with 5mM ammonium acetate in methanol. Samples were diluted with either 1:1 methanol:water for the UPLC analysis (4:1) or running solvent (a proprietary mixture provided by Biocrates) for flow injection analysis (20:1).

A study pool sample was created (5041 SPQC) by taking an equal volume from each study sample. The pooled sample was prepared and analyzed in the same way as the study samples in triplicate. On the kit plate, the SPQC was prepared in triplicate; one of these preparations was analyzed in triplicate while the other two were analyzed in a staggered manner before, during, and after the study samples in order to measure the performance of the assay across the sample cohort. The five analyses of this pool can be used to assess potential quantitative drift across the analysis of the plate, or in larger studies, to assess batch effects.

UPLC separation of amino acids and biogenic amines was performed using a Waters (Milford, MA) Acquity UPLC with a Waters Acquity 2.1 mm x 50 mm 1.7 μm BEH C18 column fitted with a Waters Acquity BEH C18 1.7 μm Vanguard guard column. Analytes were separated using a gradient from 0.2% formic acid in water, to 0.2% formic acid in acetonitrile. Total UPLC analysis time was approximately 7 minutes per sample. Acylcarnitines, sphingolipids, and glycerophospholipids were analyzed by flow injection analysis (FIA) with total analysis time of approximately 3 minutes per sample. Using electrospray ionization in positive mode, samples for both UPLC and flow injection analysis were introduced directly into a Xevo TQ-S triple quadrupole mass spectrometer (Waters) operating in the Multiple Reaction Monitoring (MRM) mode. MRM transitions (compound-specific precursor to product ion transitions) for each analyte and internal standard were collected over the appropriate retention time. The UPLC-MS/MS data were imported into Waters application TargetLynx for peak integration, calibration, and concentration calculations. The UPLC-MS/MS data from TargetLynx and FIA-MS/MS data were analyzed using Biocrates MetIDQ software. For statistical comparisons of glycerophospholipids and sphingolipids, a Wilcoxon rank-sum test was performed. All other tests were a student’s t-test.

For the energy metabolites including Acetyl-CoA, NAD+, Glutathione, cAMP, AMP, ADP, and ATP, an alternate assay was performed on the same cell pellet following the sonication step. The samples were then placed in a cold aluminum sample block on dry ice and incubated for 10 minutes. Next the samples were centrifuged for 10 minutes at 4°C and 15,000 g and stored at -80°C until ready for analysis. The samples were warmed to 4°C on ice and centrifuged again for 10 minutes at 4°C and 15,000 g to pellet any solids. Forty microliters of supernatant from each sample was pipetted into a glass total recovery vial (Waters) labeled with its corresponding DPMSR ID number. The remaining pellet from each sample was stored at -80°C. A study pool quality control (SPQC) sample was prepared by combining 5 μL of supernatant from each sample into a 1.5 mL tube (Eppendorf). Stable Isotope Labeled (SIL) standard material, the 13C Credentialed E. coli kit (MS-CRED-KIT) was purchased from Cambridge Isotope Laboratories. This is an E. coli extract from uniformly 13C-labeled E. coli. The material was tested to have minimal to no contributing signal in the light channel, using injections of only the 13C-labeled standard in previous experiments. Nine hundred microliters (900 μL) of sample resuspension solvent was created by taking one vial of 13C-labeled MS-CRED-KIT containing 100 μL of lysate and adding 400 μL of 80:20 v/v methanol/water. This resuspension solution was prepared immediately before addition to the samples. This solution was also used as the internal standard blank during the analysis. Ten microliters of the 13C-labeled *E*. *coli* resuspension solution was added via repeater pipette to each sample in the glass total recovery vials. Four microliters from each sample was injected for analysis by LC-MS/MS.

Liquid chromatographic separation was performed using a Waters Acquity UPLC with a 2.1 mm x 30 mm, 1.7 μm pore size ethylene bridged hybrid (BEH) amide column (Waters PN: 186004839). Mobile phase A was composed of water with 10 mM ammonium hydrogen carbonate (AmBic) (Millipore Sigma, St. Louis, MO) containing 0.2% ammonium hydroxide (NH4OH) generated as follows: 3.34 mL of 30% ACS grade NH4OH was added to 1 L water, followed by the addition of 0.3982 g AmBic. Mobile phase B was neat acetonitrile (Optima LCMS grade Thermo). The weak needle wash was mobile phase B and the strong needle wash was mobile phase A. The total length of the LC Gradient Program is 5.00 minutes. The outlet of the analytical column was connected directly via electrospray ionization into a Xevo TQ-S mass spectrometer (Waters) with positive/negative mode switching. Retention time scheduling with 30 second windows was used to minimize concurrent MRM transitions, and automatic dwell calculation was used to maximize dwell time while maintaining at least 8 points across the chromatographic peak. Eighty milliseconds (80 msec) was set as the polarity-switching delay. Positive and negative ion electrospray were alternated during the entirety of an LC gradient program for one injection. In ESI+ mode, capillary voltage was 3.0kV, source offset was 50V, desolvation temperature was 400°C, desolvation gas flow was 650 L/hr N2, cone gas was 150 L/hr N2, and nebulizer pressure of 7.0 bar was used. Source parameters for ESI- ionization were the same as ESI+, with the exception of the capillary voltage was set to -2.0 kV. Each sample was analyzed in Multiple Reaction Monitoring (MRM) mode in the mass spectrometer during the LC gradient program as ions eluted from the LC column.

### Statistics

In all figures, *P ≤ 0.05, **P ≤ 0.01, ***P ≤ 0.001 and ns *P* > 0.05. Error bars represent s.e.m. unless otherwise indicated. *In vivo* comparisons were made by Wilcoxon rank-sum tests. All other *P* values were calculated using a two-tailed student’s t-test unless otherwise noted. For HAPTRIG tool statistics, 1,000 genome-wide permutation simulations were used and *P* values from Wilcoxon rank-sum comparisons to these permutations were used to calculate significant differences from expected pathway perturbations caused by randomly permuted gene sets.

## Supporting information

S1 Fig Examples with CAIRN. **A,** CAIRN tested for co-amplifications of the oncogenes *MYC* and *PTK2* (*PTK2* is better known by its encoded oncoprotein FAK). **B,** Pie charts of CAIRN amplification findings. Coincident CNAs do not always dominate exclusive CNAs of oncogenes, however, co-amplification is also common on the same chromosome arm.
(TIF)

S2 Fig LC3B genetic changes in ovarian (OV) and breast (BRCA) TCGA tumors. CAIRN was used to quantify and display copy-number alterations in serous ovarian cancer and breast cancer cohorts studied by the TCGA. All CNA-available tumors are shown in the top panels, whereas those with corresponding SNV data are shown in the bottom panels, with CAIRN markings for tumor suppressors (black) and oncogenes (green) in patients with the indicated CNA event.
(TIF)

S3 Fig Clinical and genetic characteristics associated with autophagy gene loss. **A,** Racial group proportion data are plotted for patients with primary tumors containing a loss in one of *LC3B* or *BECN1*, both, or neither. A fisher’s exact test was performed on the White racial group against all other groups, with a *P* > 0.05 indicated by “ns”. **B,** Similarly, Stage data were tested for differences. In the fisher’s exact test, the largest group (stage III) was tested against all other groups. All comparisons were *P* > 0.05, “ns”. **C,** The age at diagnosis were compared by Wilcoxon rank-sum test, with *P* > 0.05 indicated by “ns”. **D,** Somatic mutation counts were compared by Wilcoxon rank-sum test. **E,** Percent genome altered per tumor group were compared to the “neither” group by Wilcoxon rank-sum test, with **P ≤ 0.01. Boxplot error bars represent furthest outliers. **F,** KmPlot outputs of human SOC tumors with or without at least one loss of the *BECN1* gene, the *MAP1LC3B* gene, or either gene. **G,** KmPlot outputs of human SOC tumors with high or low expression of the indicated autophagy genes. **H,** Kaplan-Meier plot of TCGA SOC (OV) tumors analyzed by HAPTRIG for the autophagy pathway, with low and high levels of pathway scores separated by tertiles.
(TIF)

S4 Fig Copy-number profiles of common ovarian cancer cell lines. Segmented data were downloaded from the UCSC Xena Browser for the CCLE and NCI-60 lines. Displayed are CNAs visualized by IGV. For reference, TCGA OV tumors are also displayed.
(TIF)

S5 Fig Acidic organelles have impaired turnover with autophagy gene knockdown. **A,** SKOV3 cells were tested for accumulation of AO following treatment of an autophagy inducer (Rapa, rapamycin), an autophagosome clearance inhibitor (CQ, chloroquine), or both, for 4 h. **B,** Quantitation of the microscopy data shown in (A). **C-D,** Similar tests as in (A,B) with IGROV1 cells.
(TIF)

S6 Fig Metabolomics with autophagy gene knockdowns. **A,** Lysate immunoblots from three independently created, passaged, and pelleted SKOV3 cells containing lentiviral incorporation of the indicated shRNAs. Lysates immunoblotted were from the identical samples as those submitted for metabolomics analysis. N = 6 per condition, from three experiments with two biological replicates. **B,** Quantitation of the immunoblots. **C-G,** Individual metabolites were compared to shScr controls. *P ≤ 0.05, and error bars represent s.e.m. **H,** Cell lysate immunoblots of SKOV3 cells and a clone modified by CRISPR-Cas9 to eliminate *LC3B*. **I,** Comparison of all shown metabolites between shBECN1 and shLC3B averages with a linear correlation model shown. **J,** Comparison of all shown metabolites between Cas9-knockout *LC3BΔ* and shLC3B averages with a linear correlation model shown.
(TIF)

S7 Fig Unaffected oncogenic phenotypes. **A,** Scratch wound migration assay of confluent IGROV1 cells. Note the slower timeline compared to SKOV3 cells. Quantitation includes N = 8 replicates from two independent experiments. **B,** A crystal violet growth assay confirmed trends in (A) were not due to enhanced growth rate. Shown is a representative experiment of two independent experiments, with four biological replicates. **C,** SKOV3 cells transduced with the corresponding shRNAs were tested by alkaline comet assay for ssDNA and dsDNA breaks. N > 50 cells per condition, from three independent assays. **D,** SKOV3 cells knocked down for LC3B or BECN1 were tested for centrosome size abnormalities by γ-Tubulin staining. N > 100 cells per condition, from two independent assays. **E,** Immunoblot of SKOV3 and IGROV1 cells transduced with *BECN1* targeting shRNA. The neighboring gene *BRCA1* was tested for alterations in protein levels. **F,** IGROV1 cells were imaged for γH2AX puncta. N > 1100 cells from two independent assays.
(TIF)

S8 Fig Autophagy knockdown increases focal and megabase CNAs. **A,** Genomic DNA from the 30 passage SKOV3 cells from was profiled using high-density Oncoscan arrays and analyzed for copy-number changes (Fig 4). Copy-number alterations (CNAs) were quantified for each sample by size. Genome-wide CNAs were summed and graphed for each biological replicate. *P ≤ 0.05, **P ≤ 0.01, ***P ≤ 0.001, by Wilcoxon rank-sum test. **B,** CNA counts for individual chromosomes are displayed.
(TIF)

S9 Fig *Becn1*+/- tumor morbidity. Complements Fig 6, the MISIIR Large-T-Antigen (TAg+) ovarian cancer mouse model. **A,** Littermate mice were euthanized according to morbidity: either difficulty moving, 20% weight loss, or development of ascites. While there was a trend, *Becn1*+/- TAg and littermate control *Becn1*+/+ TAg mice did not have statistically significant differences in cancer-related morbidity with the sample number tested. **B,** Copy-number analysis for the *Brca1* region of TAg+ tumors with or without *Becn1* heterozygous deletion. No CNA deletions overlapped *Brca1* in the four tumors tested from each group.
(TIF)

S10 Fig Whole-genome copy-number alteration plots of murine tumors. Terminal tumors shown in Fig 6 were harvested for genomic DNA and processed on an Illumina HiSeq4000 for whole-genome DNA reads. Data were controlled for GC content and mappability. In addition, each tumor’s DNA was then normalized to control normal tissue: adjacent uterus. Copy-number was determined by HMMcopy, using 500kb windows (containing 1000–2000 reads per window). A custom R script was used to use HMMcopy outputs and plot the visual copy-number changes shown here. Red indicates a gain of magnitude 0.2 or more (log2 units), blue indicates a loss of magnitude 0.2 or more.
(TIF)

S1 Table Genes altered by CNAs in the SKOV3 spontaneous evolution experiment. Each sample is tabulated for its log2 ratio copy-number difference from the mean of all four shScr samples. Additionally, a second sheet describes which genes were affected by gene-breakage wherein a CNA end occurs within the coding region. HAPTRIG pathway information is tabulated here.
(XLS)

S2 Table Proteins which interact with BECN1 are enriched for chromosome organization and sister chromatid cohesion genes. BioGrid was used as a resource for protein-protein interactions. GO term enrichment was tested by the GOrilla online tool (cbl-gorilla.cs.technion.ac.il) using all human genes found in BioGrid as a background list.
(XLS)

S3 Table Segmented data and gene-level analysis of CNAs found in the mouse tumor DNA experiments. Each sample is tabulated for its log2 ratio copy-number difference as determined by HMMcopy.
(XLS)
