# Supplementary figures and images for: Autophagy gene haploinsufficiency drives chromosome instability, increases migration, and promotes early ovarian tumors
Source: PLoS Genet. 2020 Jan 10;16(1):e1008558. doi: 10.1371/journal.pgen.1008558 (PMC6953790; doi:10.1371/journal.pgen.1008558)

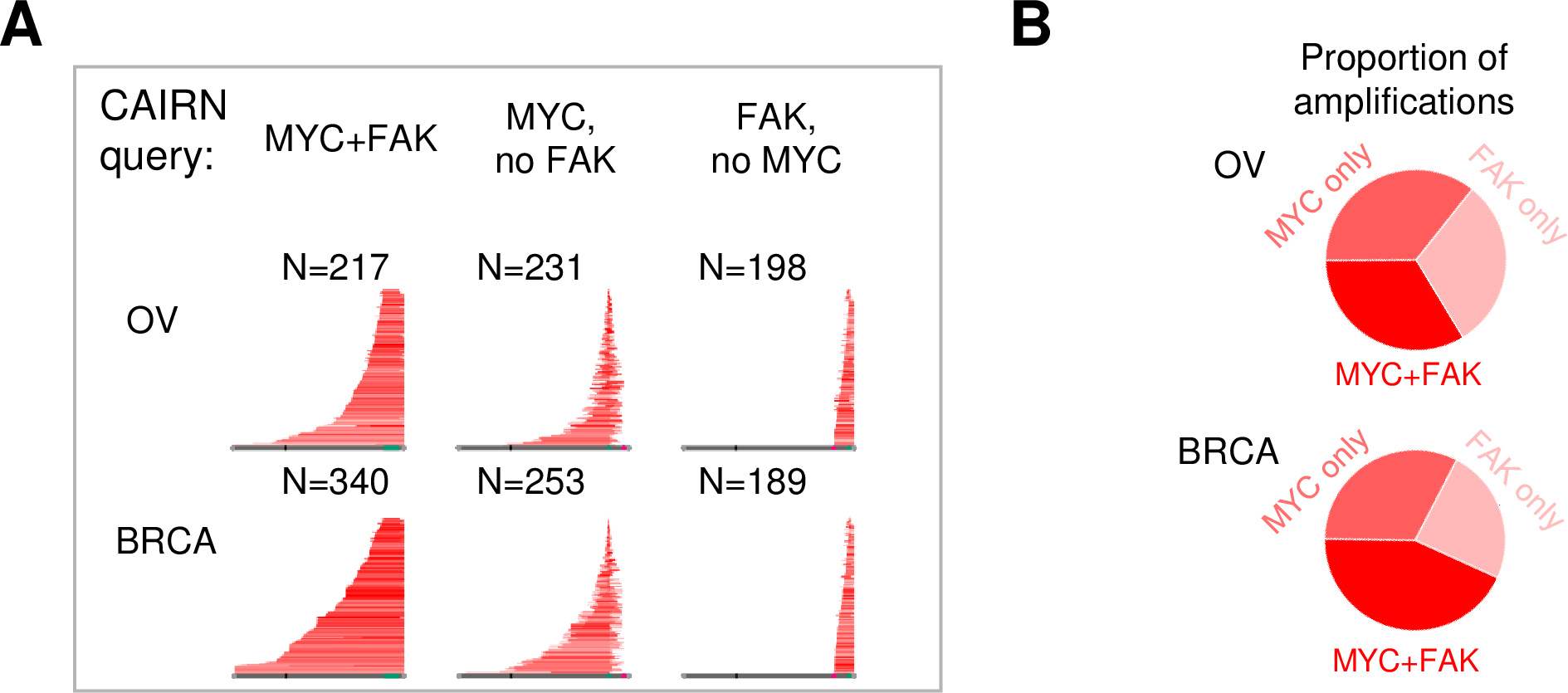

Supplement: S1 Fig — A, CAIRN tested for co-amplifications of the oncogenes MYC and PTK2 (PTK2 is better known by its encoded oncoprotein FAK). B, Pie charts of CAIRN amplification findings. Coincident CNAs do not always dominate exclusive CNAs of oncogenes, however, co-amplification is also common on the same chromosome arm. (TIF) [file pgen.1008558.s001.tif]

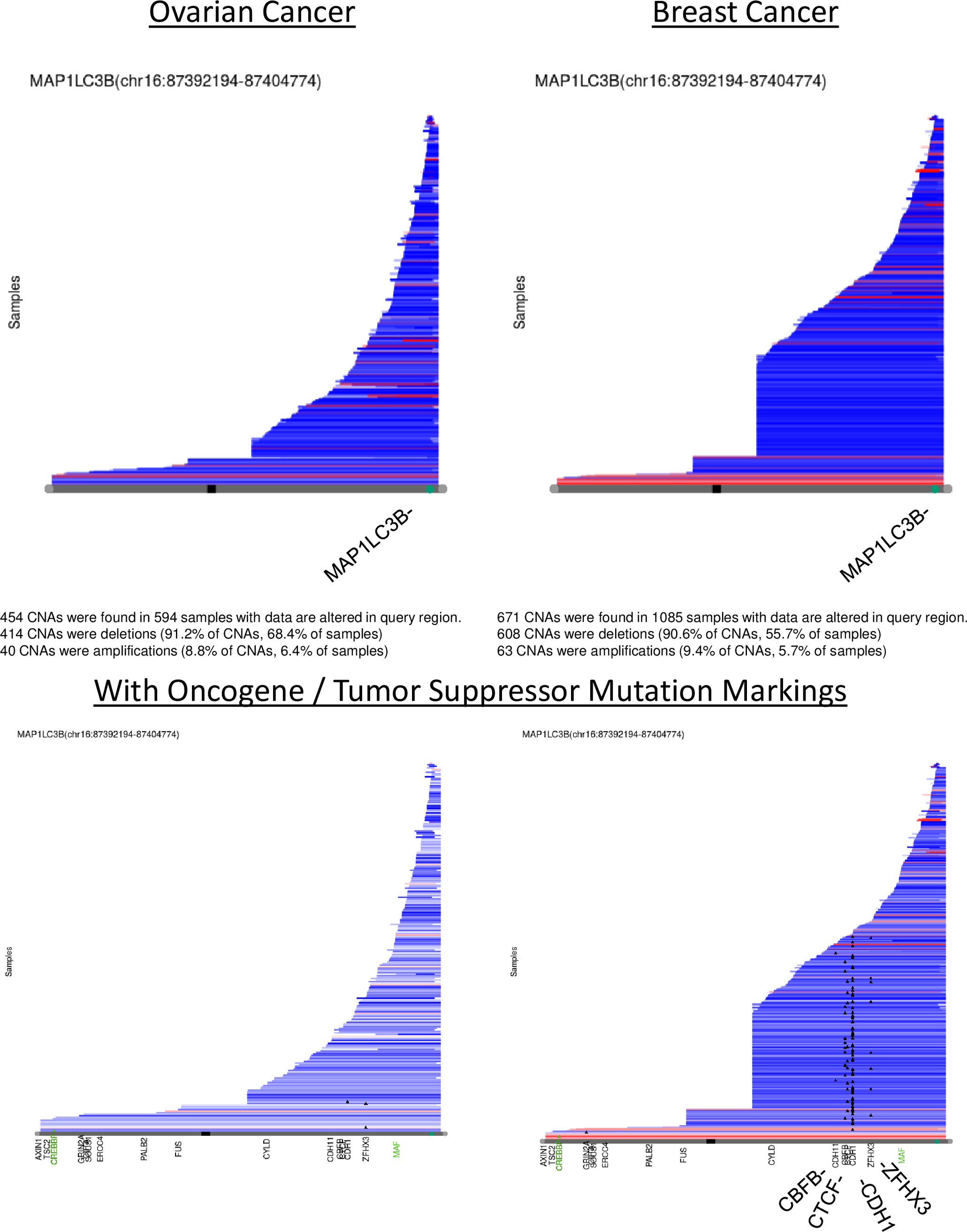

Supplement: S2 Fig — CAIRN was used to quantify and display copy-number alterations in serous ovarian cancer and breast cancer cohorts studied by the TCGA. All CNA-available tumors are shown in the top panels, whereas those with corresponding SNV data are shown in the bottom panels, with CAIRN markings for tumor suppressors (black) and oncogenes (green) in patients with the indicated CNA event. (TIF) [file pgen.1008558.s002.tif]

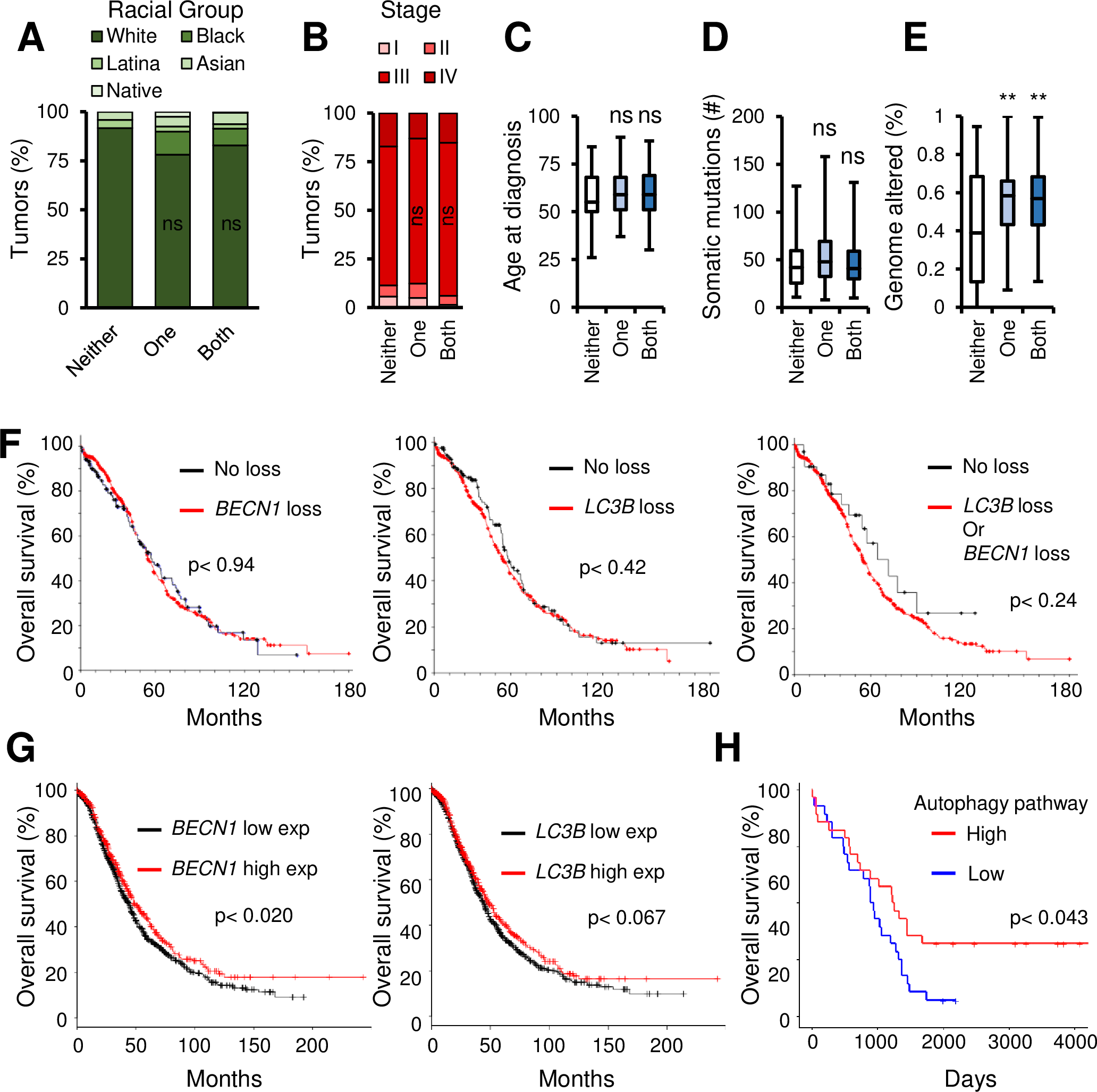

Supplement: S3 Fig — A, Racial group proportion data are plotted for patients with primary tumors containing a loss in one of LC3B or BECN1, both, or neither. A fisher’s exact test was performed on the White racial group against all other groups, with a P > 0.05 indicated by “ns”. B, Similarly, Stage data were tested for differences. In the fisher’s exact test, the largest group (stage III) was tested against all other groups. All comparisons were P > 0.05, “ns”. C, The age at diagnosis were compared by Wilcoxon rank-sum test, with P > 0.05 indicated by “ns”. D, Somatic mutation counts were compared by Wilcoxon rank-sum test. E, Percent genome altered per tumor group were compared to the “neither” group by Wilcoxon rank-sum test, with **P ≤ 0.01. Boxplot error bars represent furthest outliers. F, KmPlot outputs of human SOC tumors with or without at least one loss of the BECN1 gene, the MAP1LC3B gene, or either gene. G, KmPlot outputs of human SOC tumors with high or low expression of the indicated autophagy genes. H, Kaplan-Meier plot of TCGA SOC (OV) tumors analyzed by HAPTRIG for the autophagy pathway, with low and high levels of pathway scores separated by tertiles. (TIF) [file pgen.1008558.s003.tif]

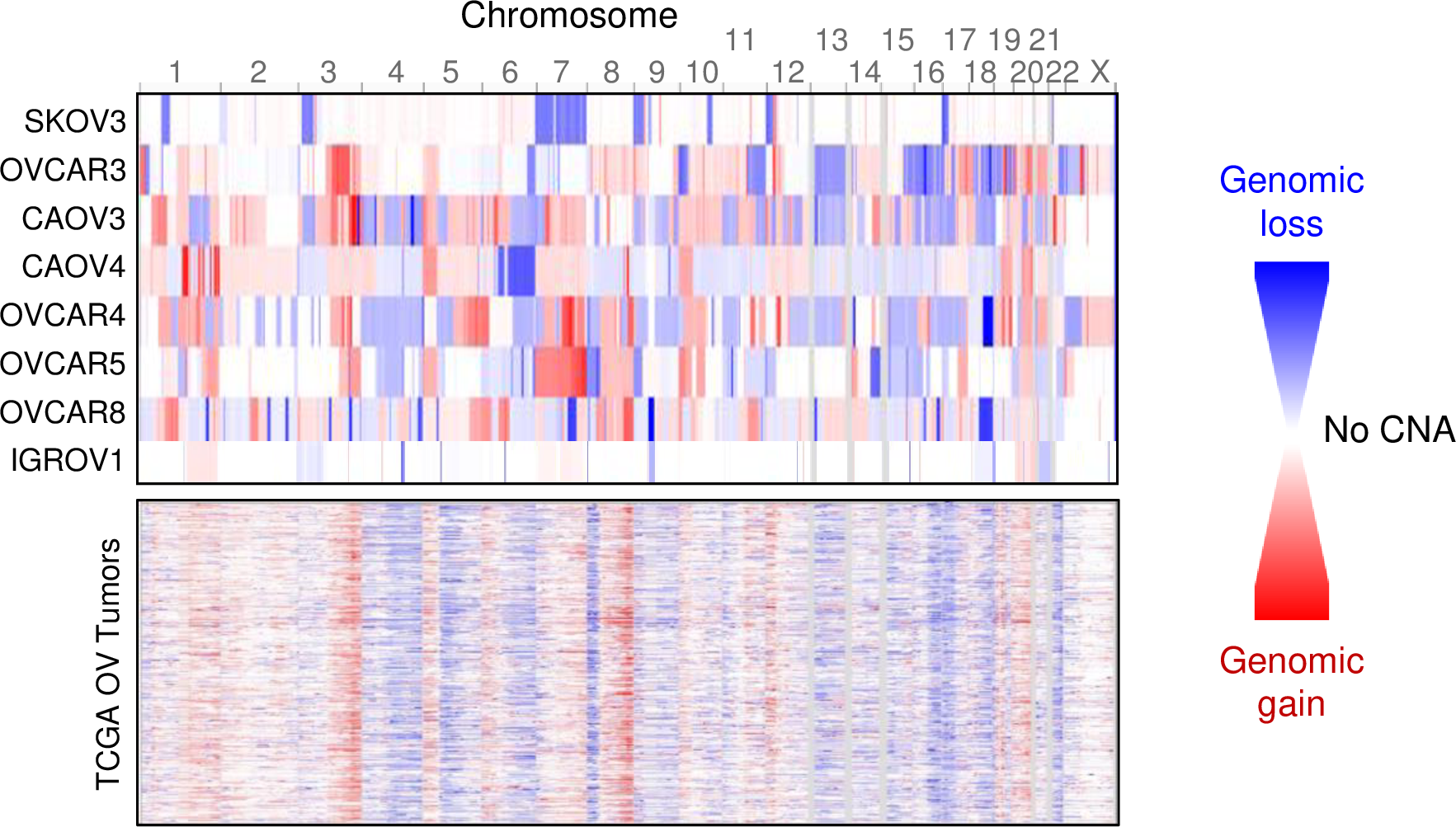

Supplement: S4 Fig — Segmented data were downloaded from the UCSC Xena Browser for the CCLE and NCI-60 lines. Displayed are CNAs visualized by IGV. For reference, TCGA OV tumors are also displayed. (TIF) [file pgen.1008558.s004.tif]

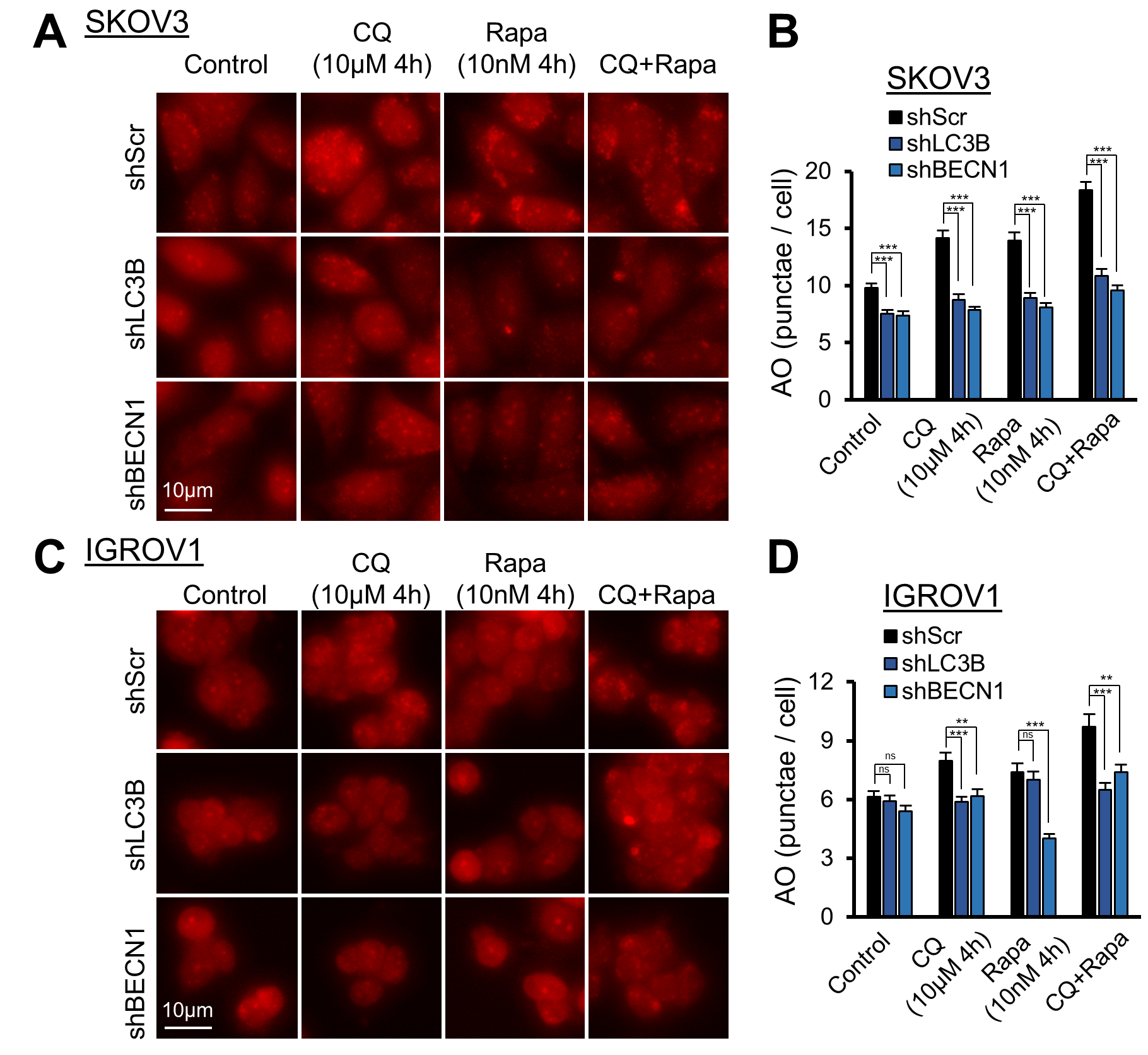

Supplement: S5 Fig — A, SKOV3 cells were tested for accumulation of AO following treatment of an autophagy inducer (Rapa, rapamycin), an autophagosome clearance inhibitor (CQ, chloroquine), or both, for 4 h. B, Quantitation of the microscopy data shown in (A). C-D, Similar tests as in (A,B) with IGROV1 cells. (TIF) [file pgen.1008558.s005.tif]

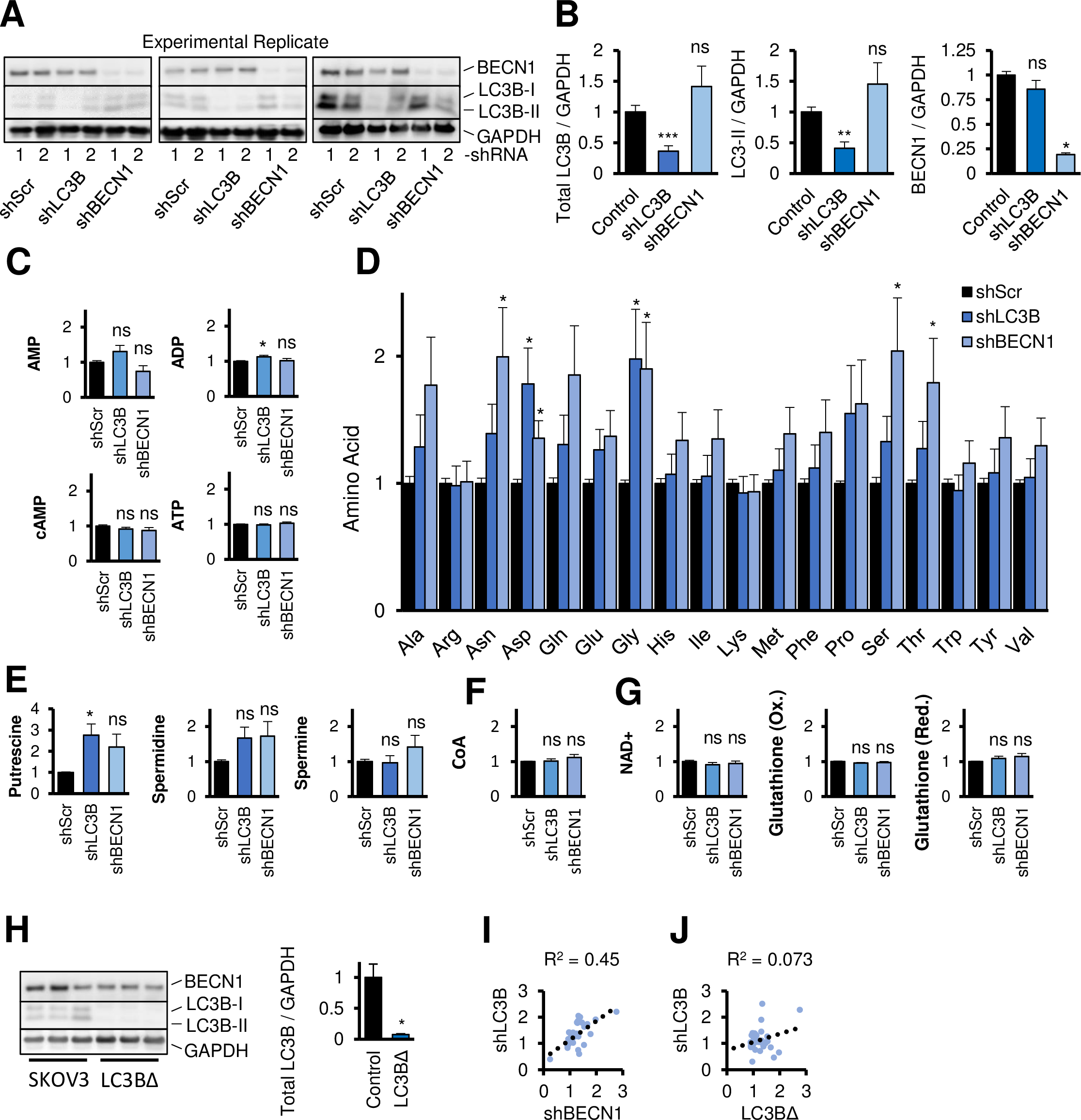

Supplement: S6 Fig — A, Lysate immunoblots from three independently created, passaged, and pelleted SKOV3 cells containing lentiviral incorporation of the indicated shRNAs. Lysates immunoblotted were from the identical samples as those submitted for metabolomics analysis. N = 6 per condition, from three experiments with two biological replicates. B, Quantitation of the immunoblots. C-G, Individual metabolites were compared to shScr controls. *P ≤ 0.05, and error bars represent s.e.m. H, Cell lysate immunoblots of SKOV3 cells and a clone modified by CRISPR-Cas9 to eliminate LC3B. I, Comparison of all shown metabolites between shBECN1 and shLC3B averages with a linear correlation model shown. J, Comparison of all shown metabolites between Cas9-knockout LC3BΔ and shLC3B averages with a linear correlation model shown. (TIF) [file pgen.1008558.s006.tif]

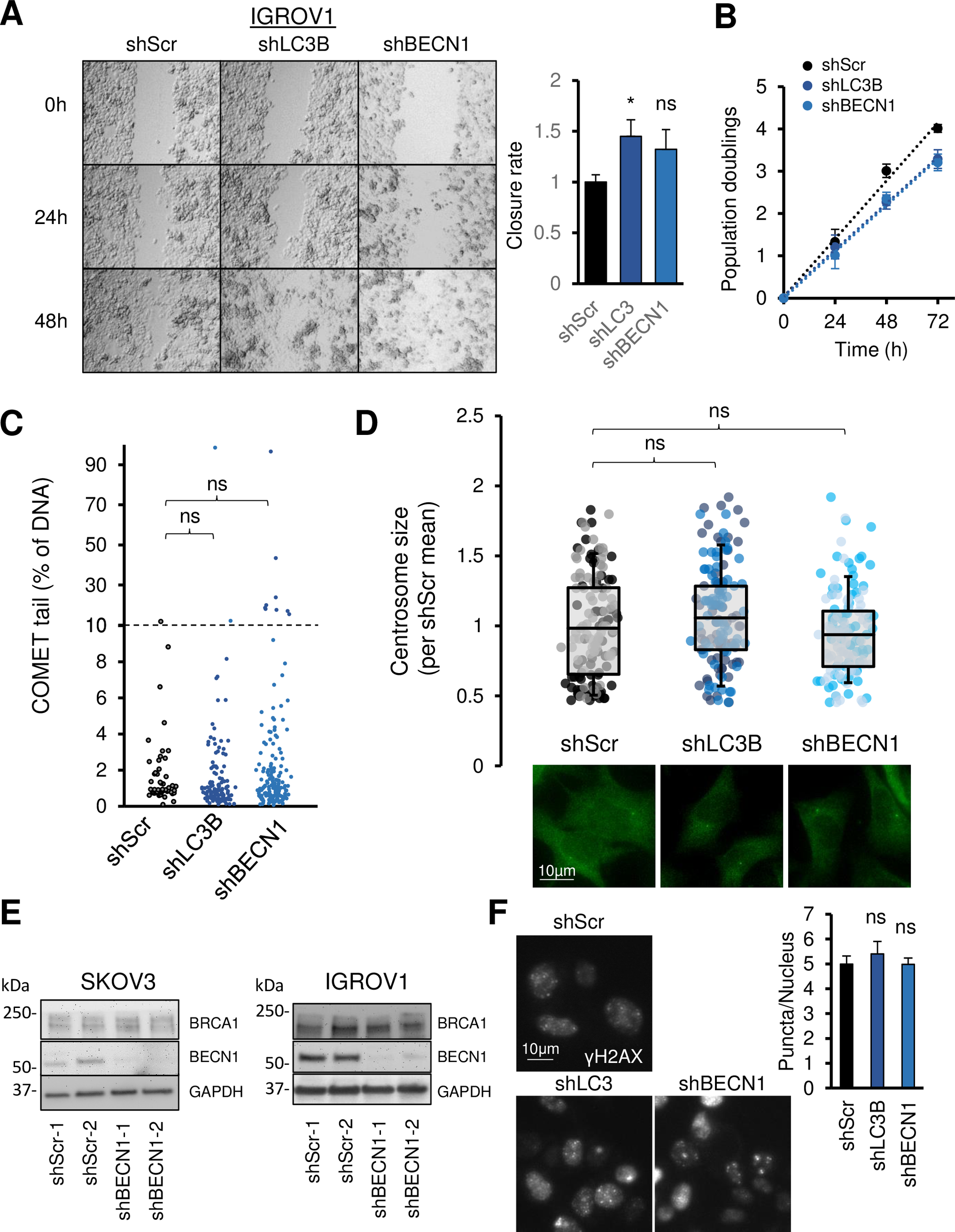

Supplement: S7 Fig — A, Scratch wound migration assay of confluent IGROV1 cells. Note the slower timeline compared to SKOV3 cells. Quantitation includes N = 8 replicates from two independent experiments. B, A crystal violet growth assay confirmed trends in (A) were not due to enhanced growth rate. Shown is a representative experiment of two independent experiments, with four biological replicates. C, SKOV3 cells transduced with the corresponding shRNAs were tested by alkaline comet assay for ssDNA and dsDNA breaks. N > 50 cells per condition, from three independent assays. D, SKOV3 cells knocked down for LC3B or BECN1 were tested for centrosome size abnormalities by γ-Tubulin staining. N > 100 cells per condition, from two independent assays. E, Immunoblot of SKOV3 and IGROV1 cells transduced with BECN1 targeting shRNA. The neighboring gene BRCA1 was tested for alterations in protein levels. F, IGROV1 cells were imaged for γH2AX puncta. N > 1100 cells from two independent assays. (TIF) [file pgen.1008558.s007.tif]

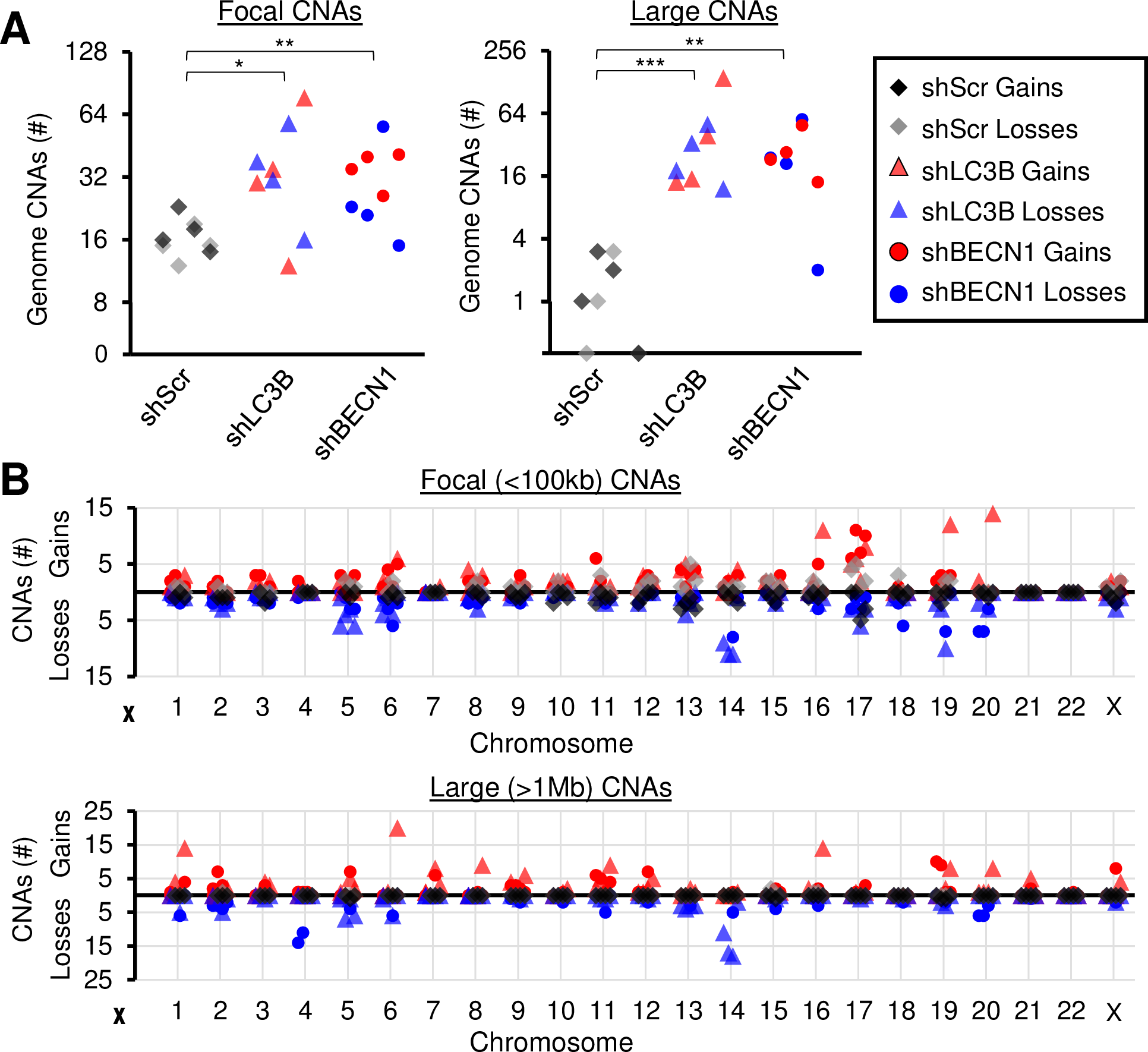

Supplement: S8 Fig — A, Genomic DNA from the 30 passage SKOV3 cells from was profiled using high-density Oncoscan arrays and analyzed for copy-number changes (Fig 4). Copy-number alterations (CNAs) were quantified for each sample by size. Genome-wide CNAs were summed and graphed for each biological replicate. *P ≤ 0.05, **P ≤ 0.01, ***P ≤ 0.001, by Wilcoxon rank-sum test. B, CNA counts for individual chromosomes are displayed. (TIF) [file pgen.1008558.s008.tif]

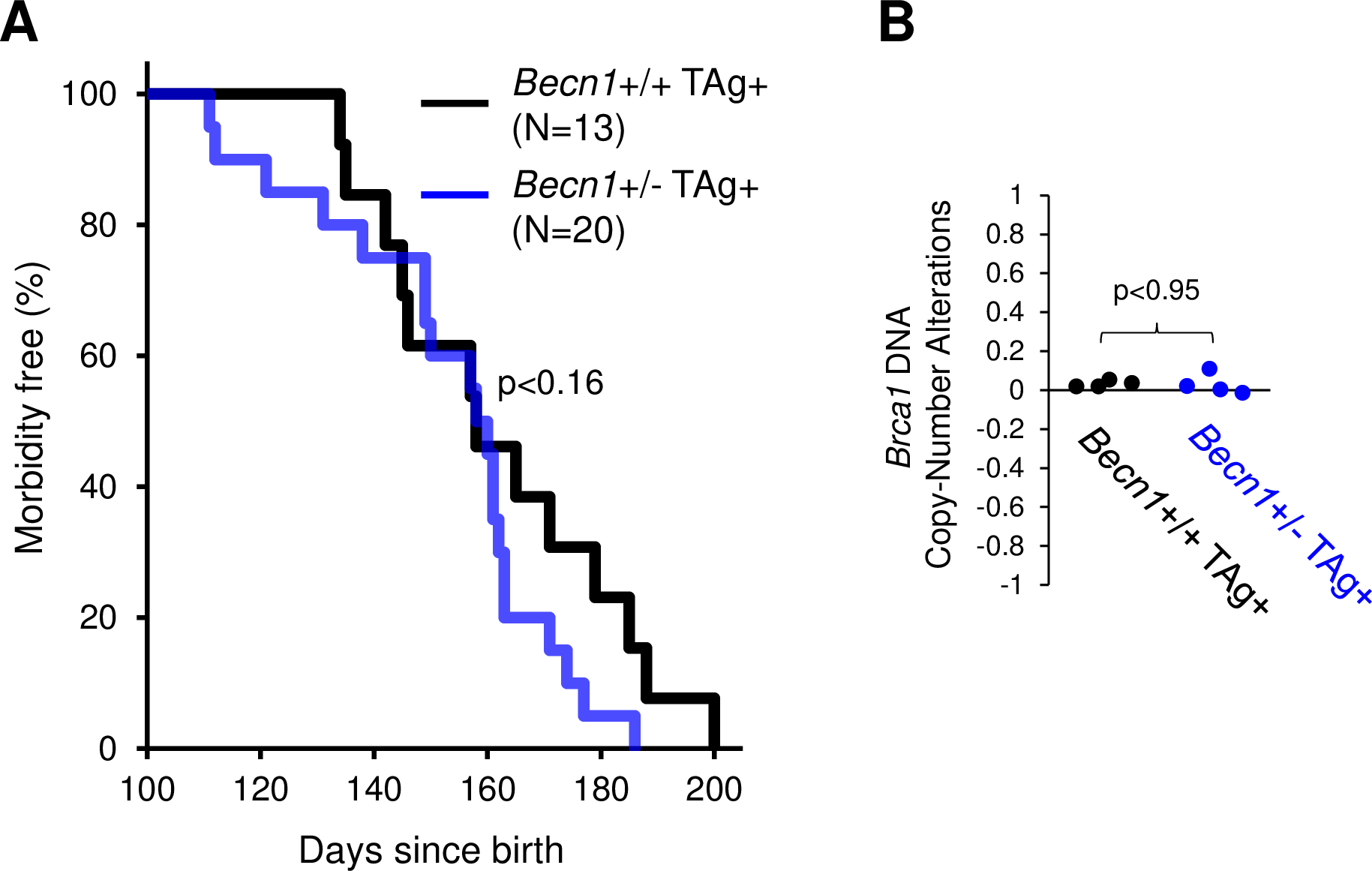

Supplement: S9 Fig — Complements Fig 6, the MISIIR Large-T-Antigen (TAg+) ovarian cancer mouse model. A, Littermate mice were euthanized according to morbidity: either difficulty moving, 20% weight loss, or development of ascites. While there was a trend, Becn1+/- TAg and littermate control Becn1+/+ TAg mice did not have statistically significant differences in cancer-related morbidity with the sample number tested. B, Copy-number analysis for the Brca1 region of TAg+ tumors with or without Becn1 heterozygous deletion. No CNA deletions overlapped Brca1 in the four tumors tested from each group. (TIF) [file pgen.1008558.s009.tif]

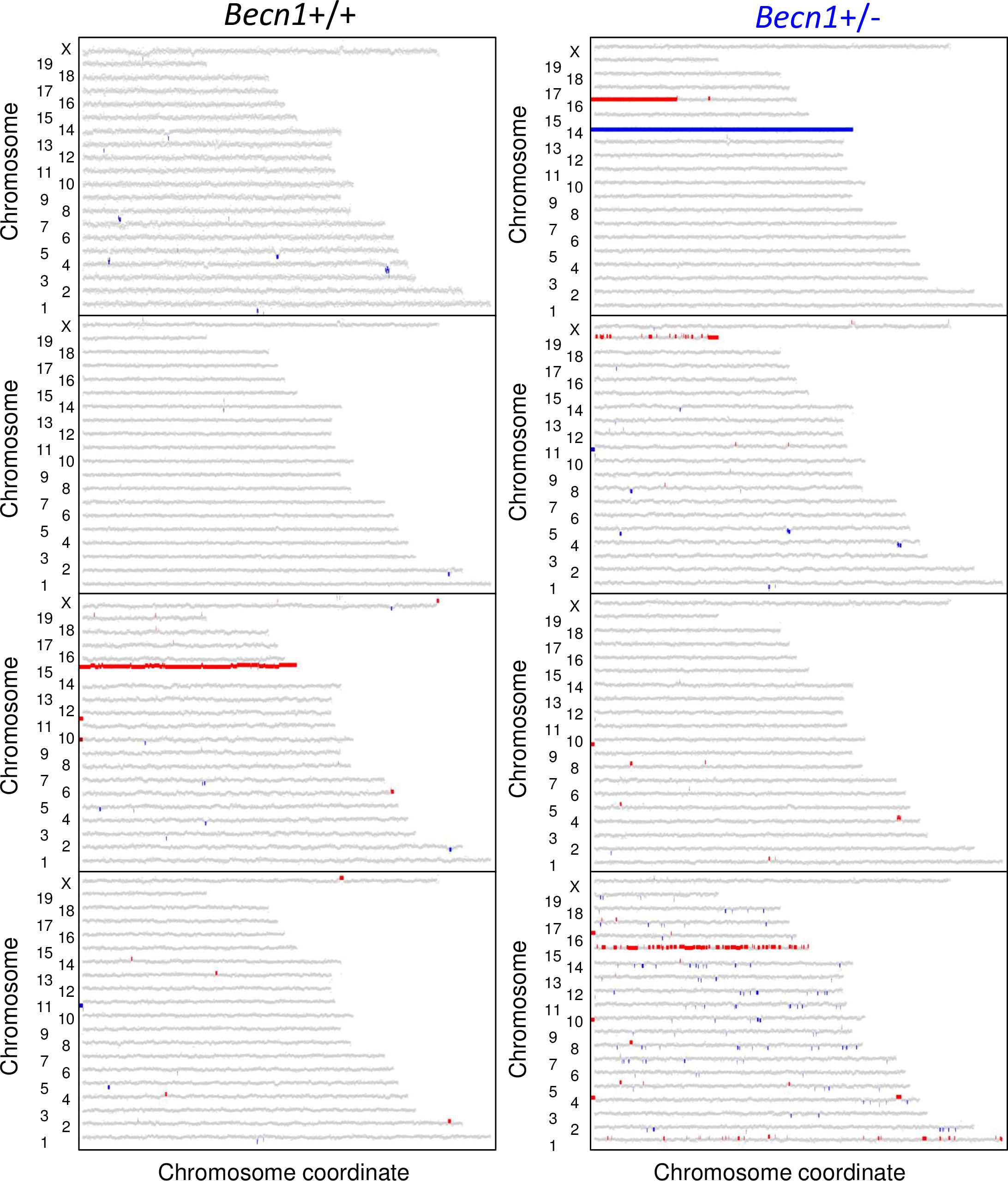

Supplement: S10 Fig — Terminal tumors shown in Fig 6 were harvested for genomic DNA and processed on an Illumina HiSeq4000 for whole-genome DNA reads. Data were controlled for GC content and mappability. In addition, each tumor’s DNA was then normalized to control normal tissue: adjacent uterus. Copy-number was determined by HMMcopy, using 500kb windows (containing 1000–2000 reads per window). A custom R script was used to use HMMcopy outputs and plot the visual copy-number changes shown here. Red indicates a gain of magnitude 0.2 or more (log2 units), blue indicates a loss of magnitude 0.2 or more. (TIF) [file pgen.1008558.s010.tif]
